# Combining machine learning with Cox models to identify predictors for incident post-menopausal breast cancer in the UK Biobank

**DOI:** 10.1038/s41598-023-36214-0

**Published:** 2023-06-07

**Authors:** Xiaonan Liu, Davide Morelli, Thomas J. Littlejohns, David A. Clifton, Lei Clifton

**Affiliations:** 1 Nuffield Department of Population Health, University of Oxford, Oxford, UK; 2 Big Data Institute, University of Oxford, Old Road Campus, Oxford, OX3 7LF UK; 3 Department of Engineering Science, University of Oxford, Oxford, UK

**Keywords:** Biochemistry, Cancer, Genetics, Biomarkers, Diseases, Medical research

## Abstract

We aimed to identify potential novel predictors for breast cancer among post-menopausal women, with pre-specified interest in the role of polygenic risk scores (PRS) for risk prediction. We utilised an analysis pipeline where machine learning was used for feature selection, prior to risk prediction by classical statistical models. An “extreme gradient boosting” (XGBoost) machine with Shapley feature-importance measures were used for feature selection among $\approx$ 1.7 k features in 104,313 post-menopausal women from the UK Biobank. We constructed and compared the “augmented” Cox model (incorporating the two PRS, known and novel predictors) with a “baseline” Cox model (incorporating the two PRS and known predictors) for risk prediction. Both of the two PRS were significant in the augmented Cox model ($p<0.001$). XGBoost identified 10 novel features, among which five showed significant associations with post-menopausal breast cancer: plasma urea (HR = 0.95, 95% CI 0.92–0.98, $p<0.001$), plasma phosphate (HR = 0.68, 95% CI 0.53–0.88, $p=0.003$), basal metabolic rate (HR = 1.17, 95% CI 1.11–1.24, $p<0.001$), red blood cell count (HR = 1.21, 95% CI 1.08–1.35, $p<0.001$), and creatinine in urine (HR = 1.05, 95% CI 1.01–1.09, $p=0.006$). Risk discrimination was maintained in the augmented Cox model, yielding C-index 0.673 vs 0.667 (baseline Cox model) with the training data and 0.665 vs 0.664 with the test data. We identified blood/urine biomarkers as potential novel predictors for post-menopausal breast cancer. Our findings provide new insights to breast cancer risk. Future research should validate novel predictors, investigate using multiple PRS and more precise anthropometry measures for better breast cancer risk prediction.

## Introduction

Breast cancer is the most common cancer among women, with 2.3 million women diagnosed with breast cancer in 2020^1^. Decades of efforts have established multiple predictors^2^ for the disease, including reproductive factors^3–5^, lifestyle^6,7^, and inherited genetic factors^8–10^. Despite the identification of multiple modifiable predictors, breast cancer remains a leading cause of death, with 685,000 deaths in 2020 worldwide. Pre- and post-menopausal breast cancers are usually regarded as etiologically different^11–15^.

Traditionally, predictor discovery for diseases such as breast cancer is hypothesis-driven. While it is reasonable to use classical statistical models (e.g. logistic regression) to assess these predictors, some novel predictors may be overlooked in the discovery stage in information-rich data prior to constructing a classical prediction model. Machine learning (ML) methods are able to handle both a large number of predictors and complex non-linear relationships, hence may provide assistance in the discovery of predictors^16,17^. Previous ML studies have primarily focused on how ML approaches compare to conventional models for breast cancer risk prediction cancer^18–22^, but there are a lack of studies on utilising ML for predictor identification. The increasing availability of large and detailed cohorts, such as the UK Biobank (UKB), offer the opportunity to utilise hypothesis-free approaches for the identification of potentially novel predictors.

Recent years have witnessed the rapid development of polygenic risk scores (PRS) which aggregate the effect of a large number (e.g. hundreds or thousands) of genetic variants associated with a specific disease or trait, identified using genome-wide association (GWAS) studies. PRS have been proposed in a variety of clinical practice and research, including providing better future disease risk and identifying people at high risk for targeted treatment or screening strategies^23^. For example, PRS added benefits in identifying populations that would most benefit from statin prescription^24–26^; PRS added accuracy to existing coronary artery disease risk predictors (e.g. Framingham risk score)^27^; and breast cancer PRS have been incorporated into existing risk prediction models such as the Breast and Ovarian Analysis of Disease Incidence and Carrier Estimation Algorithm (BOADICEA)^28^ and Tyrer-Cuzick model^29^.

Interactions between PRS and phenotypic features (e.g. gene-environment interactions) have been suggested with breast cancer and its subtypes, including alcohol consumption, height, hormone therapy^30^, family history^31^, hormonal birth control use, menopausal status^32^, and use of corticosteroids^33^. However, the overall evidence is inconsistent.

Our analysis pipeline consists of ML methods for feature selection, followed by classical Cox models for risk prediction. Our intention was neither to seek superiority among different approaches, nor to build competing prediction models for breast cancer. Our goal was to demonstrate that ML methods can be used for feature selection to complement classical statistical methods. Furthermore, we used SHapley Additive exPlanation (SHAP) feature dependence plots to explore potential interactions between PRS and phenotypic features. We also provided necessary statistical considerations before constructing classical Cox models to further investigate the potentially novel features selected by ML methods.

## Methods

### Study design and participants

The UKB is a large-scale population-based prospective cohort with detailed phenotypic and genetic data from over half a million participants recruited between 2006 and 2010 across 22 assessment centres in England, Wales and Scotland^34^. The baseline data were collected in person via questionnaires, verbal interview with a trained nurse, physical examinations and biological samples. Follow-up information was obtained through linkage to electronic medical records of death and cancer registries and hospital inpatient records. The UKB study received ethical approval from the North West Multi-center Research Ethics Committee (REC reference: 11/NW/03820). All participants gave written informed consent before enrolment in the study, which was conducted in accordance with the principles of the Declaration of Helsinki.

**Figure 1:**
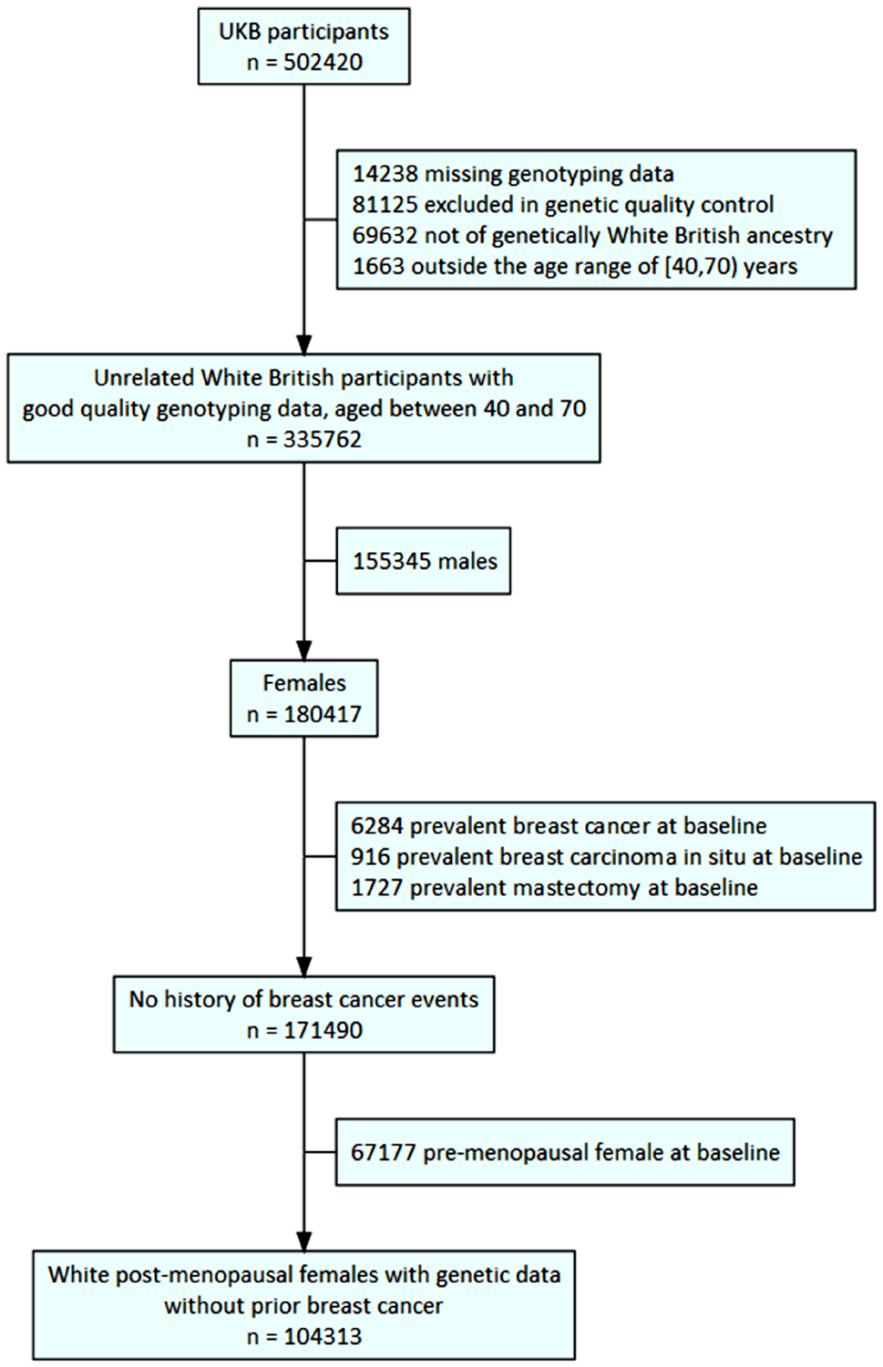
Flowchart illustrating the selection process for our study population.

In this study, we focused only on post-menopausal women due to the etiological heterogeneity of breast cancer by menopause status^11–15^. We restricted our study population to a sub-cohort of UKB female participants who were post-menopausal with age 40–69 at baseline, met UKB internal genetic quality control (UKB field 22020), were of genetic White ancestry (UKB field 22006), and had no history of breast cancer, breast carcinoma in situ or mastectomy at baseline (Fig. 1).

Our final study population was further randomly divided into training (80%) and test (20%) sets (i.e. training-test split) for subsequent ML analyses.

### Prevalent and incident post-menopausal breast cancer

Prevalent breast cancer cases were identified using International Classification of Diseases codes (ICD-9: 174.X and ICD-10: C50.X) from the linked cancer registry data, with the date of breast cancer diagnosis preceding or on the date of baseline assessment.

Incident cases were ascertained longitudinally using cancer registry data, supplemented by record-level hospital inpatient data due to the reporting delay in registries. The follow-up time for each participant was calculated as the number of years from the date of baseline assessment until the earliest of the following: breast cancer diagnosis, date of death from other causes, date of loss to follow-up, date of mastectomy or last date of medical record availability in UKB: 28th February 2021 in England, 28th February 2021 in Scotland, and 28th February 2018 in Wales.

### Polygenic risk scores

Due to the substantial discordance in individual-level risk categorisation between different PRS for the same disease^35^, we included two breast cancer PRS as potential genetic features: PRS_313_^9^ and PRS_120k_^36^. Neither PRS used UKB data in its derivation stage, hence both are suitable for calculation within the UKB population without the concern of inflated effect estimates due to sample overlap. PRS_313_ consisted of 313 (pre-Quality control) independent (correlation $<0.9$) genetic variants associated with breast cancer, developed using hard-thresholding and stepwise forward regression with ${p<10}^{-5}$ in Breast Cancer Association Consortium (BCAC) data. PRS_120k_ consisted of 118,388 (pre-Quality control) variants, developed using the lassosum method^37^ from the same BCAC data.

We used the imputed genetic data from UKB (version 3, March 2018 release). Full details of genotyping, imputation, genetic array, and principal components (PCs) are described elsewhere^38^ and Supplementary Materials. We performed further variant Quality control (QC) checks across the whole cohort using a published pipeline^39^, excluding variants that were not available in UKB, variants poorly imputed in UKB (imputation information $<$ 0.4), ambiguous variants (A/T or C/G single nucleotide polymorphisms (SNPs) with minor allele frequency $>$ 0.49) and variants with minor allele frequency (MAF) $<$ 0.005. This led to 305 variants remaining in PRS_313_ and 115,300 in PRS_120k_ (Supplementary Table 1).

We then performed sample QC, excluding participants who were related (third degree or higher), sex discordant, or identified as outliers for genotype missingness or heterozygosity (as these could indicate poor sample quality), using sample QC data provided centrally by UKB (UKB field 22020) that retained a maximal set of unrelated individuals. Finally, we calculated the PRS as the weighted sum of effect allele dosages, and divided by the number of alleles using PLINK2^40^.

### Phenotypic features

In a phenome-wide scan of predictors for breast cancer, we considered 2,315 cross-sectional features from the UKB (Supplementary Table 2), reflecting socio-demographics, lifestyle, family history, early life and reproductive factors, blood and urine assays, physical measures, cognitive function, medication use and health conditions at baseline.

We mapped the 6,745 unique self-reported medications (UKB field 20003) to 411 distinct codes at level 4 of the Anatomical Therapeutic Chemical (ATC) classification system^33,41^. For example, if participants self-reported taking “kliofem tablet” or “kliovance 1 mg/0.5 mg tablet”, they would be categorised into the “G03FA” ATC group (i.e. “contraceptive and hormone replacement therapy (HRT) related medication” group). Since our study population is post-menopausal women only, any G03FA medication is assumed to be HRT, and is referred to as such throughout this paper.

We identified prevalent cancer diagnoses (level 2 ICD-10 codes under “Chapter II Neoplasms”, “Chapter XV Pregnancy, childbirth, and the puerperium”) from cancer registry data, and prevalent non-cancer diagnoses (level 2 ICD-10 codes except Chapters U,V,W,X,Y, and Z) from hospital inpatient data.

We did not include administrative variables, inapplicable pilot fields, male-specific factors, family history of family members of adoptees, history of surgical operations (because they likely reflect existing diagnoses that were already included as input features), and fields collected exclusively during follow-up (e.g. imaging data).

For biomarker assays, UKB has performed internal quality checks and excluded values where no data or an error was returned from the analyser, the values were outside the reportable range of the assay at the time of measurement or there was an aliquot problem. Full details of quality control were described in UKB Resource 5636. Among biomarkers, we excluded oestradiol and rheumatoid factor due to their high proportions of values below the lower reportable range.

The main pre-processing we performed on training data prior to ML analysis was assigning the following three categories as missing: “Prefer not to answer”, “Do not know”, and empty entries. We then removed features with missing rate $>$ 30%, and those where all participants had the same value (such as rare diseases which no participants were affected by at baseline) which were of no discriminative utility, yielding 1,737 input features for ML models. All features were fitted in original scale from UKB without transformations.

### Analysis pipeline

**Figure 2:**
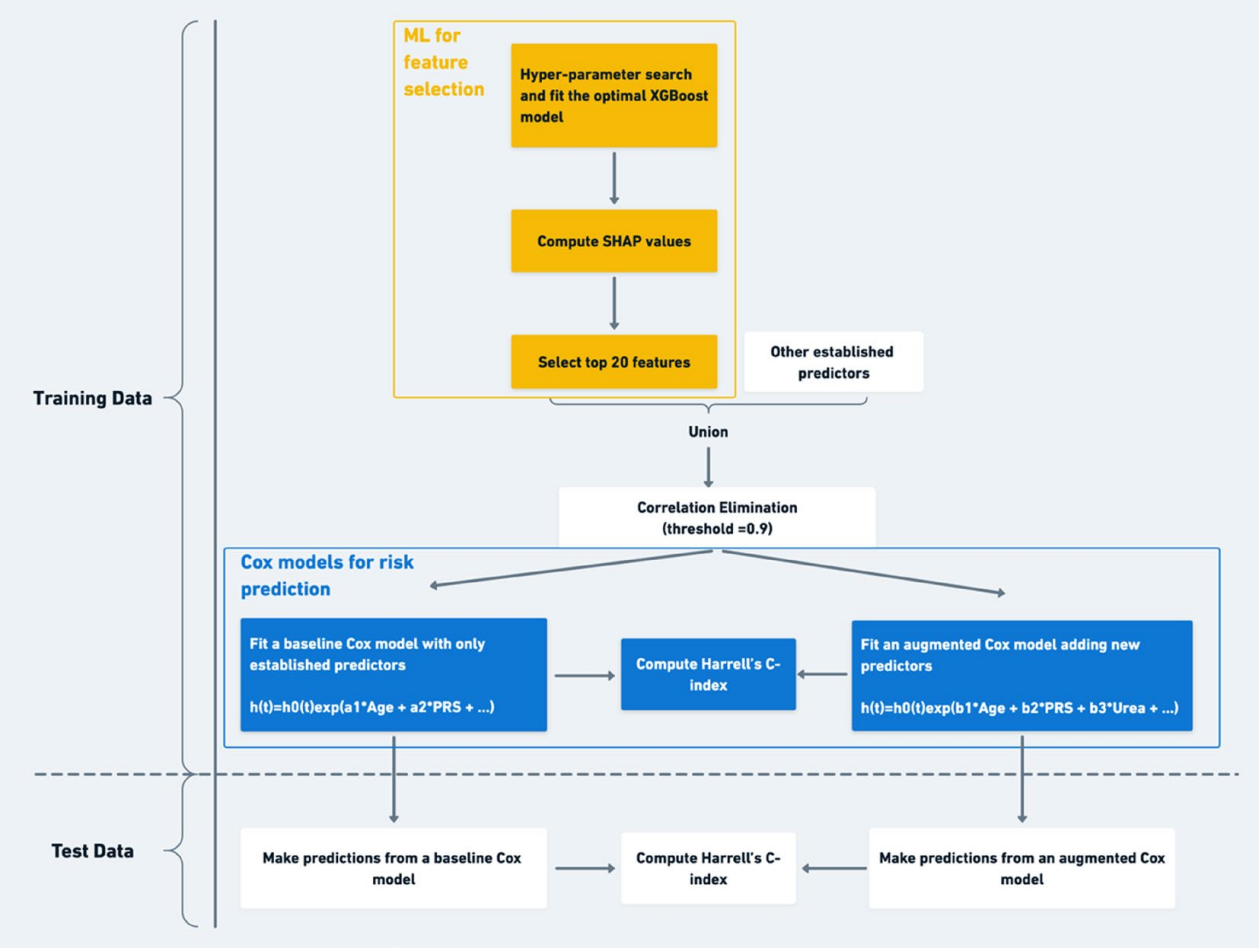
Analysis pipeline. ML models were used for predictor discovery, followed by classical Cox modelling for further investigation. ML: Machine learning. XGBoost: extreme gradient boosting machine. SHAP: Shapley Additive Explanation. PRS: Polygenic risk scores.

We adapted an analysis pipeline^16^ for combining ML and statistical approaches (Fig. 2), but with a fundamental difference in the definition of “test data”. We pre-specified the 20% test data as being unseen hold-out data that were not used in model construction, for the purpose of testing the performance of the Cox models obtained using the 80% training data (i.e. guarding against model overfitting). In contrast, Madakkatel et al. did not have such unseen hold-out test data; Cox models in the latter were constructed using their “test data” which were not held-out, whereas ours were built using the 80% training data. We believe that this separation between training and test enforces the avoidance of overfitting, and of reporting results using the same data that were visible to the construction of the model, in the standard manner for separating training and test sets.

Our further enhancements included grid search for hyper-parameter tuning and SHAP dependence plots for exploring PRS-predictor interactions. The tree-based eXtreme Gradient Boosting (XGBoost) machine learning algorithm^16^ was used to discover novel features among ≈1.7 k variables. Features of high importance according to the SHAP measure were regarded as potentially novel predictors, and were subsequently investigated by classical Cox models.

### Model-based feature selection

Feature selection is necessary prior to constructing an analytical model for risk prediction. This task can be undertaken in many ways (e.g. backwards selection, correlation analysis, Delphi process). We used model-based feature selection (via a tree-based XGBoost machine) due to its ability to rank large number of features, prior to risk prediction by classical Cox models. XGBoost machine is also able to handle missing data, and reveal non-linear relationship between features and the outcome. The intention was to obtain the “best of both worlds”, by using non-linear methods to identify candidate associated inputs and then using conventional medical statistical models for maximum interpretability of the results.

Tree-based XGBoost is an ensemble learning method in which decision trees are built in a sequential manner. After initialising the model prediction by minimising a regularised loss function, the algorithm builds each tree by minimising the residuals from prior ones. The final model prediction is the weighted sum of the predictions from these sequential trees. It is capable of revealing non-linear relationships among correlated features from large datasets in a memory-efficient manner.

Our outcome (i.e. response variable) was the time-to-event outcome of incident breast cancer which consists of a binary indicator (present/absent) and the follow-up time. The XGBoost model was trained with negative partial log-likelihood of Cox proportional hazard model (i.e. “cox-loss”) as the loss function. Missing data was regarded as containing information (i.e. missing not at random, MNAR). During the training of the model, samples with missing values were assigned a default direction in each branch to either the left or the right child node, based on the gain.

For hyper-parameter tuning, we performed grid search (Supplementary Table 3) with five-fold cross validation (CV) on training data using cox-loss as the evaluation metric. The optimal set of hyper-parameters for XGBoost were found to be: maximum depth $=$ 2, number of trees $=$ 1,635, learning rate $=$ 0.01, minimum of child weights $=$ 3, gamma $=$ 1, subsample $=$ 0.8, column sample by tree $=$ 0.8, and lambda for regularisation $=$ 20. The graphical illustration of the optimal XGBoost model is shown in Supplementary Fig. 1. The Harrell’s C-index obtained from the five-fold CV was 0.717 on training data and 0.667 on test data, indicating that the model was not over-fitted. The reduction in C-index of approximately 0.05 is in keeping with expectations for evaluating a model in-sample (on training data) to out-of-sample (on hold-out test data). To investigate the impact of different loss functions, we trained an additional XGBoost model with a binary indicator of incident breast cancer using log-loss as the loss function (Supplementary Materials).

A variety of measures exist for obtaining features of high importance, such as the XGBoost built-in methods^42,43^, permutation based feature importance^44^, and SHAP values [arXiv:1705.07874]. Existing literature^16^ [arXiv:1802.03888] suggests that SHAP values are the most consistent and stable among the above methods. These properties are vital aspects of feature selection as they provide assurance that the selected features are robust to the perturbation of input data^45^. SHAP values also have the advantages of faster computation and better visualisation compared to permutation-based methods (Supplementary Materials). Despite those advantages, SHAP values have its limitations (e.g. not suitable for causal inference) and could potentially generate misleading interpretations, which could hide biases^46,47^.

We therefore chose SHAP values as our main feature importance measure, but also implemented the XGBoost default feature importance (“weight”) and permutation-based methods for comparison. The SHAP value of each feature was first computed using one sample at a time to reflect the local effect on the sample, and then aggregated by taking the mean of absolute SHAP values (SHAP_ma_) across all samples to summarise the global attribution of this feature. In addition to the global SHAP values shown in a summary bar plot (Fig. 4), we presented the local SHAP values in an information-rich "beeswarm” plot, which shows both the relative ranking of features and the relationship of each feature with the outcome. The local SHAP values were further visualised in SHAP dependence plots^48^ to explore the potential relationship between PRS and phenotypic features.

### Statistical model

Following the ML analysis, we further examined the extracted features as follows:

We regarded the top 20 features ranked by SHAP_ma_ as “important”. The union of these 20 features with the established risk factors forms the set of potentially “important” features. We then computed different forms of pairwise correlation $r$ among these different types of features from the training data. Spearman’s rank coefficient was computed for pairs of numeric features, and Cramer’s V (computed using the Chi-squared statistic) for pairs of categorical features. The correlation between a numeric and a categorical feature was computed by regressing the numeric feature on the categorical feature and then taking the square root of the proportion of variation explained (also called correlation ratio).

Within each pair of features that was identified as highly correlated ($r>0.9$), we removed either the feature with most missing data, or the auxiliary one. This step is necessary to reduce the collinearity prior to constructing a linear (e.g. Cox) statistical model when the model will be used to draw statistical inference on the estimated effect of features.

The missingness within each feature was carefully assessed at this stage, as the number of features had now been sufficiently reduced (e.g. from over 1k to under 30) to permit such close inspection. For example, the variables “Age at first birth” and “Number of live births” needed to be considered together to ensure the imputed data were reasonable for women who have not had children. We performed multiple imputation using the *mice* package in R^49^ to impute the missing data under the assumption of missing at random (MAR). In contrast, the XGBoost machine had assigned a missing category to missing data, effectively assuming MNAR.

We note that the above statistical procedure is essential preparation before constructing classical statistical models and must not be overlooked. The analytical power of their elegant equations comes from careful attention to model specifications and thorough examination of the underlying assumptions.

Following the necessary preparation above, we constructed a Cox proportional hazard model (i.e. the augmented Cox model) using the training data to assess the associations between novel features and incident breast cancer, adjusting for established risk factors. Since PRS were present in the model, we also adjusted for genetic array (UKB field 22000) and the first 10 genetic PCs (UKB field 22009) to account for the underlying population structure.

To determine whether the novel features identified by ML improve model performance, we built a separate Cox model using the training data with only the two PRS and known risk factors (i.e. the baseline Cox model). We computed Brier score at 10-year for assessing overall model performance and Harrell’s C-index for assessing risk discrimination. We used the two sets of baseline hazard at 10-year and model coefficients (i.e. “beta values”) yielded from these two Cox models to compute the 10-year risks and prognostic index (i.e. variable $\times$ beta) of each participant in the training (80%) and test (20%) data, respectively. These 10-year risks were subsequently used to compute Brier score at 10 years and prognostic indices were used to compute Harrell’s C-index^50^ using the training and test data, respectively. Both the augmented and baseline Cox models were pre-specified in our statistical analysis plan.

The proportional hazard assumption of Cox models was visually assessed using scaled Schoenfeld residuals. Multicollinearity was assessed by computing the variance inflation factor (VIF); values less than 10 were considered acceptable. Statistical tests were two-tailed, and performed using a 5% significance level.

As sensitivity analyses, we built additional Cox models using the training data to investigate the potential “PRS $\times$ phenotypic features” interactions indicated by the SHAP dependence plots. To further assess the robustness of feature importance ranking, we implemented another machine learning model, histogram-based gradient boosting machines (GBM) inspired by LightGBM^51^ (Supplementary Table 4, Supplementary Fig. 3, and Supplementary Materials).

XGBoost version 1.5.0 and SHAP version 0.40.0 were implemented in Python version 3.8.8 and Cox model analyses were conducted using R version 4.0.2.

## Results

### Participants characteristics

Baseline characteristics of the study population are presented in Table 1. Of the 104,313 participants included in our study, 4,010 (3.8%) developed breast cancer over the median follow-up of 11.9 (IQR 11.0–12.6) years. The 80% training and 20% test sets had 3,252 and 758 incident cases of breast cancer, respectively.

**Table 1:** Baseline characteristics of the study population by incident breast cancer status (N = 104,313). Median (interquartile ranges, IQR) are presented for continuous variables, frequency (percentage) are reported for categorical variables. Percentages may not add up to 100 due to rounding. N is the number of non-missing values. Note*: Both PRS were multiplied by the number of alleles in the score for easy comparison. PRS: Polygenic risk scores. BrCa: Breast Cancer. BMI: Body mass index. HRT: hormone replacement therapy. ML: machine learning. MET: Metabolic Equivalent Task. U/L: units per litre.

	Without incident BrCa (n = 100,303)	With incident BrCa (n = 4,010)	N
$PR{S}_{120k}$*	−0.14 (−0.32, 0.03)	−0.02 (−0.19, 0.16)	104,313
$PR{S}_{313}$*	−0.42 (−0.83, −0.01)	−0.13 (−0.54, 0.27)	104,313
**Established risk factors**			
Testosterone, nmol/L	0.97 (0.69, 1.33)	1.03 (0.75, 1.43)	81,362
Age at recruitment, years	61.30 (57.08, 64.91)	61.83 (58.00, 65.14)	104,313
Age at menopause, years	50.00 (48.00, 53.00)	51.00 (48.00, 54.00)	97,540
Alcohol units per week	6.00 (0.00, 12.00)	6.00 (0.00, 13.50)	104,313
IGF-1, nmol/L	20.16 (16.56, 23.71)	20.40 (16.64, 23.89)	98,843
Age at first birth (Categorical)			
No Births	16,164 (16.1%)	643 (16.0%)	16,807
< 20	7616 (7.6%)	278 (6.9%)	7894
20–30	60,526 (60.4%)	2371 (59.2%)	62,897
30–40	15,181 (15.2%)	685 (17.1%)	15,866
> = 40	690 (0.7%)	30 (0.7%)	720
BMI, kg/m^2^	26.12 (23.54, 29.55)	26.78 (24.12, 30.32)	103,958
Family history of BrCa	10,807 (10.8%)	621 (15.5%)	104,313
Summed MET minutes per week	1786.00 (834.00, 3546.00)	1667.25 (797.50, 3336.38)	79,981
HRT user	2257 (2.3%)	159 (4.0%)	104,313
Age at menarche, years	13.00 (12.00, 14.00)	13.00 (12.00, 14.00)	101,470
Number of live births	2.00 (1.00, 2.00)	2.00 (1.00, 2.00)	104,261
**Novel predictors selected by ML**			
Plasma urea, mmol/L	5.35 (4.61, 6.18)	5.31 (4.57, 6.17)	99,337
Basal metabolic rate, KJ	5489.00 (5138.00, 5912.00)	5598.00 (5226.00, 6021.00)	102,593
Plasma phosphate, mmol/L	1.21 (1.12, 1.31)	1.20 (1.10, 1.29)	90,431
Sodium in urine, mmol/L	55.20 (35.50, 83.70)	57.20 (37.73, 86.00)	100,733
Red blood cell count, 10^12/L	4.34 (4.12, 4.55)	4.37 (4.15, 4.58)	101,089
Aspartate aminotransferase, U/L	23.80 (20.80, 27.60)	23.60 (20.60, 27.20)	99,091
Creatinine (enzymatic) in urine, mcmol/L	5530.00 (3412.00, 9027.00)	5872.00 (3622.50, 9612.00)	101,036
Gamma glutamyltransferase, U/L	22.20 (16.80, 33.20)	23.30 (17.45, 35.50)	99,365
Alkaline phosphatase, U/L	86.60 (73.10, 102.30)	86.45 (72.90, 101.60)	99,411
C-reactive protein, mg/L	1.42 (0.69, 2.95)	1.61 (0.79, 3.29)	99,211

### Input features for ML

**Figure 3:**
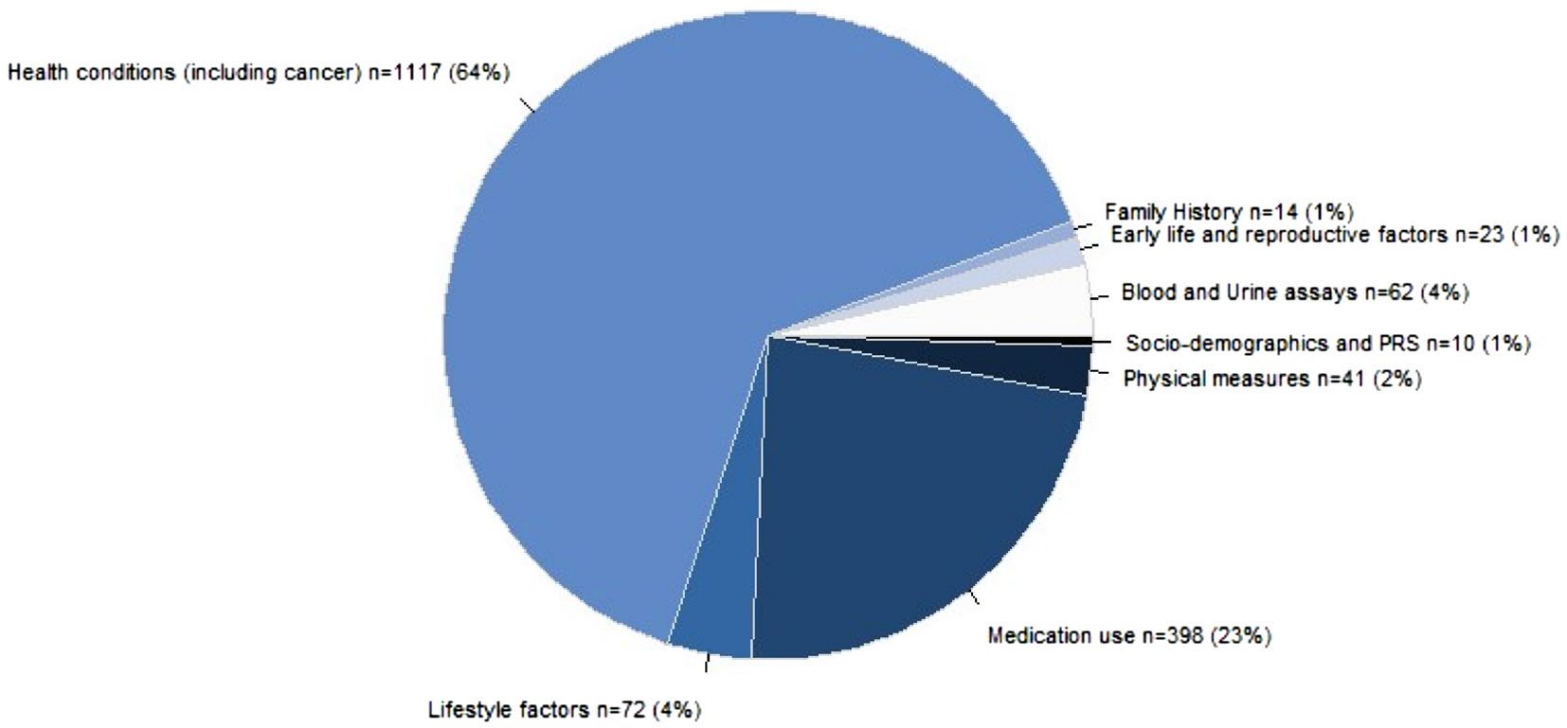
Categories of the 1,737 input features for ML analyses; n (%) represents the number (percent) of features included in each category.

Figure 3 shows the categories of the 1,737 input features for the XGBoost ML models, over half of which were in the “Health conditions” category (e.g. infectious diseases, circulatory diseases, and cancers). “Lifestyle factors” include alcohol, diet, and sleep. “Medication use” includes medication for blood pressure control, and birth control (further detail in Supplementary Table 2). “Physical measures” include blood pressure, arterial stiffness, and anthropometry. “Socio-demographics” include age, education, employment, and deprivation index. “Blood and urine assays” include blood counts and biochemistry (e.g. cholesterol). “Early life and reproductive factors” include birth weight and age at menarche. “Family history” includes illnesses of father, mother, and siblings. Out of 1,737 features, there were 1,590 categorical or binary features, and 147 continuous features.

### Model-based feature selection

**Figure 4:**
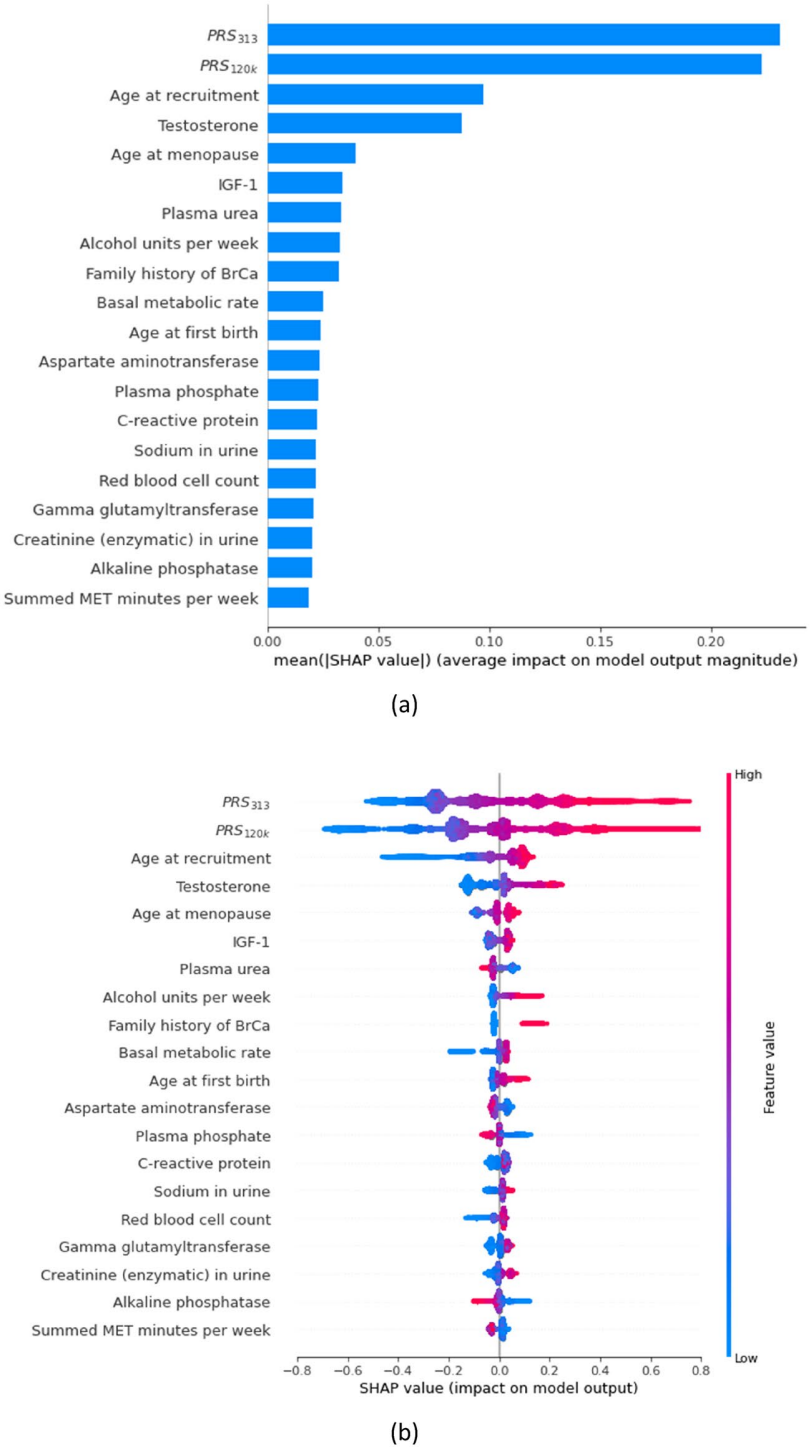
SHAP summary plots according to the XGBoost machine: (**a**) bar plot showing global SHAP values, and (**b**) beeswarm plot showing local SHAP values. The top 20 features for the risk of breast cancer were shown. Noticeably, both BrCa PRS are deemed of much higher importance than the remaining phenotypic features. PRS: Polygenic risk scores. BrCa: Breast Cancer. SHAP: SHapley Additive explanation. mean(|SHAP value|): mean absolute SHAP value, SHAP_ma_. Features in both bar plot and beeswarm plot were ranked by “SHAP_ma_”, hence their rankings are identical. In the SHAP beeswarm plot, each point represents an individual in the training data. The x-axis corresponds to the SHAP value, with vertical dispersion/jitter indicating a high density of points. The colour gradient indicates the relative magnitude of each feature (not SHAP values) with red indicating high values of the feature (e.g. older age) and blue (e.g. younger age) the opposite.

The top 20 features ranked by the highest mean absolute SHAP values (SHAP_ma_) are shown in Fig. 4 (full list of SHAP_ma_ in Additional Supplementary Table).

The SHAP beeswarm plot shows the impact of the underlying values of each feature on model outputs, which is the change in the relative hazard (i.e. ${e}^{prognostic index}$) of developing breast cancer. It is informative in three ways: (1) the ranking indicates the relative importance of features; (2) the gradient of colour indicates the direction of effects; (3) the dispersion of points indicates whether the relationship of each feature with the outcome is linear (details below).

There are a total of 14 established risk factors available in the UKB. Two of these are PRS_120k_ and PRS_313_, occupying the top two positons, with noticeably higher feature importance values than the remaining features, indicating that both PRS warrant inclusion in the subsequent models. Eight of these (age, testosterone, age at menopause, IGF-1, alcohol intake, family history of breast cancer, age at first birth, physical activity (i.e. summed metabolic equivalent task minutes per week)) appeared in the top 20 features. The four risk factors not present/ranked in the top 20 are use of HRT (ranked 26, SHAP_ma_ $=$ 0.015), parity (ranked 72, SHAP_ma_ $=$ 0.005), age at menarche (ranked 74, SHAP_ma_ $=$ 0.005), and body mass index (BMI) (ranked 79, SHAP_ma_ $=$ 0.004).

Among the top 20 ranked features, our XGBoost machine discovered novel predictors of breast cancer from the following categories:

- Body composition by impedance (UKB Category 100009) (basal metabolic rate)
- Blood count (UKB Category 100081) (red blood cell count)
- Blood biochemistry (UKB Category 17518) (plasma urea, plasma phosphate, aspartate aminotransferase, alkaline phosphatase, C-reactive protein, gamma glutamyltransferase)
- Urine assays (UKB Category 100083) (sodium in urine, creatinine in urine)

The anthropometric measure, basal metabolic rate (measured using a Tanita BC418MA body composition analyser), showed expected correlation with BMI ($r=0.73$), which is also the highest pair-wise correlation among the list of top 20 features and other established predictors. We retained basal metabolic rate in the subsequent Cox models, because these correlations are below the threshold of 0.9. Full list of features with an absolute correlation of more than 0.5 was shown in Supplementary Table 5.

The colour gradient in the SHAP beeswarm plot indicates that there were five features (plasma urea, aspartate aminotransferase, plasma phosphate, alkaline phosphatase, and physical activity) that had higher values (i.e. red points) with negative SHAP values (i.e. decrease in relative hazard). This suggests potential protective effect of these features on developing breast cancer (i.e. higher values are associated with lower risk of breast cancer).

The symmetry in the range of the positive and negative SHAP values in the SHAP beeswarm plot suggests a linear relationship between the feature and the outcome. If the range of the positive SHAP values are longer than that of the negative SHAP values (or vice versa), this may suggest a non-linear relationship.

For example, the SHAP values of alkaline phosphatase have similar lengths in the ranges of the positive and negative SHAP values, indicating a linear relationship with the outcome. We observed similar patterns for the two PRS. In contrast, age at recruitment occupies a longer range in the negative SHAP values than the positive, suggesting that younger age may have had stronger impact on breast cancer than older age (i.e. non-linear effect). To further investigate the non-linear effect of age, we performed post-hoc analyses by fitting two Cox models: (1) an unadjusted Cox model that included age only; and (2) a Cox model that further added a spline term of age with 3 inner knots at 25th, 50th, and 75th quantiles. We then performed likelihood-ratio test on the spline terms ($p<0.001$), which showed statistically significant non-linear effect of age.

During the training of our XGBoost machine, hyper-parameter tuning indicated that allowing each tree to grow down to two levels yielded the best model performance, which in turn allowed the discovery of two-way interactions among features. Given our particular interest in PRS, we used SHAP dependence plots to visualise the effect modification of PRS by each of the top 20 features (i.e. interaction PRS $\times$ predictor) (Supplementary Fig. 5–6). The SHAP dependence plots revealed potential effect modifications but with some unexpected patterns, which required further investigations before drawing inference on effect modifications. Full results and investigation are presented in Supplementary Fig. 7–9, and Supplementary Materials.

The top 20 features obtained from XGBoost with cox-loss were largely consistent with those obtained from XGBoost with log-loss (Supplementary Fig. 2). Besides the slight change in the feature importance ranking, monocytes count and whole body fat mass were ranked on the borderline by cox-loss at 21 and 30, respectively. In comparison, they were ranked at 17 and 18 by log-loss.

### Statistical analysis

Our final augmented multivariable Cox model consists of the 14 established risk factors available in the UKB (including the two PRS), the 10 potentially novel features identified by SHAP value rankings, genetic array, and the first 10 genetic PCs. Among these 10 novel features, the following five had a statistically significant association with breast cancer in post-menopausal women: basal metabolic rate, red blood cell count, plasma urea, plasma phosphate, and creatinine in urine. Among these five features, blood biochemistry features (i.e. plasma urea and plasma phosphate) were inversely associated with risk of developing breast cancer, whereas other novel features (i.e. basal metabolic rate, red blood cell count, and creatinine in urine) were positively associated. The direction of effect was consistent with the trend observed in SHAP beeswarm plot.

The remaining five novel features that did not reach the 5% significance level are: sodium in urine, aspartate aminotransferase, gamma glutamyltransferase, alkaline phosphatase, and C-reactive protein.

**Figure 5:**
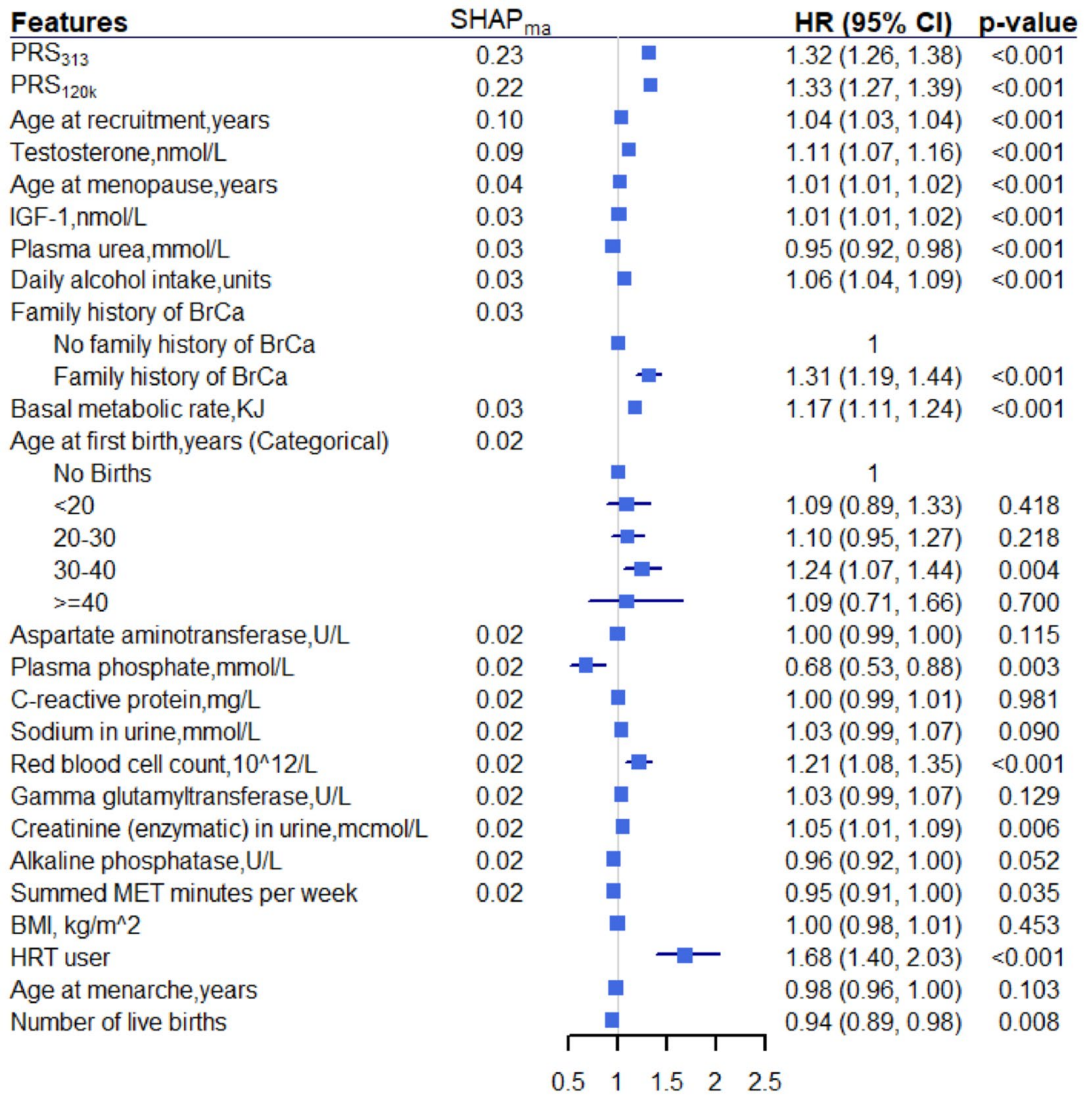
Results obtained from the augmented multivariable Cox model ranked by SHAP_ma_. The bottom four features are established risk factors that are outside the top 20 features by SHAP_ma_. Alcohol intake was scaled from weekly intake to daily intake for easy interpretation and direct comparison with existing literature. Both PRS, basal metabolic rate, sodium in urine, gamma glutamyltransferase, creatinine in urine, alkaline phosphatase, and summed MET minutes per week were standardised using the mean and standard deviation within each imputed dataset, hence the corresponding HR represents per 1 standard deviation increase. For other continuous variables, HR represents per 1 unit increase. Genetic array and first 10 PCs were adjusted in the model but omitted from the figure. PRS: Polygenic risk scores. SHAP: SHapley Additive explanation. SHAP_ma_: mean absolute SHAP value. BrCa: Breast Cancer. HR: hazard ratio. CI: confidence interval. BMI: Body mass index. HRT: hormone replacement therapy. MET: Metabolic Equivalent Task. U/L: units per litre.

Figure 5 shows the hazard ratios (HR) with 95% confidence interval and p-values of covariates in the augmented Cox model. As a pre-specified sensitivity analysis, we constructed the baseline Cox model that contains only the two PRS and the 12 established risk factors using the training data (Supplementary Table 6).

The Brier scores of baseline and augmented Cox models using the training data were both 0.032, and those using the test data were both 0.031. In the training data, Harrell’s C-index increased from 0.667 (the baseline Cox model) to 0.673 (the augmented Cox model) when adding the additional novel features selected by ML. In the test data, the corresponding Harrell’s C-index increased from 0.664 to 0.665, computed using the model coefficients obtained from the training data.

As mentioned in Sect. 3.6, the Harrell’s C-index of the XGBoost model was 0.717 from the training data (i.e. 0.044 increase from the augmented Cox model) and 0.667 (i.e. 0.002 increase from the augmented Cox model) from the test data. This increase in C-index could be due to the higher complexity of XGBoost (e.g. incorporates non-linearity) compared to classical Cox models. To interrogate the possibility of reverse causation, we conducted another sensitivity analysis to exclude the first two years of follow-up for blood and urine biomarkers ($N=\mathrm{82,491}$). We did not find evidence of reverse causation (Supplementary Fig. 4).

We additionally fitted an augmented Cox model using the 20% test data to further investigate the features discovered by the ML step (Supplementary Table 7), as an extra sensitivity analysis. We observed attenuations in associations for novel features, but the overall direction of results remained consistent with the main analyses. This could be due to both reduced sample size and possibility of chance findings.

## Discussion

The aim of this study was to demonstrate the utility of combining machine learning with traditional statistical approaches in the domain of cancer epidemiology. We identified several known risk factors (e.g. age, testosterone, and age at menopause) and five potentially novel predictors (e.g. blood counts, blood biochemistry and urine biomarkers). These latter novel features remained robustly associated with incident post-menopausal breast cancer after incorporating known risk factors in classical Cox models. Further research is necessary to confirm whether these novel predictors are potential modifiable risk factors for breast cancer, particularly the biological mechanisms of these associations. We intended to identify novel predictors for further exploration, rather than commenting on their potential causal nature.

### Novel predictors

Here we delineate the existing literature on the novel factors. Body composition measures were expected to be associated with breast cancer as obesity is a well-known risk factor for post-menopausal breast cancer^52^. The unexpected observation was that the XGBoost model selected a more detailed body composition measures, basal metabolic rate instead of BMI, indicating that more precise body composition measures could provide important information above and beyond BMI for predicting breast cancer. Previous studies^53–55^ have investigated anthropometric factors beyond BMI (e.g. waist to hip ratio, weight gain, waist circumference) and found positive associations with post-menopausal breast cancer. However more detailed anthropometric factors are worthy of further investigation. Our findings showed that basal metabolic rate is a significant predictor for breast cancer, contradicting previous studies that found no such association^56,57^. Our positive finding could be due to the statistical power conferred by the large sample size of UKB.

The SHAP feature importance ranking supported the associations of novel biomarkers with post-menopausal breast cancer, but little literature exists on this topic. Plasma urea, a blood biomarker related to kidney function, was reported to have null causal relationship with breast cancer^58^, but our study suggests it may be associated with breast cancer. Aspartate aminotransferase and alkaline phosphatase are both blood biomarkers related to liver function and were not previously associated with non-metastatic breast cancer^59^. In contrast, gamma glutamyltransferase was observed to have positive association with subtypes of breast cancer in the Apolipoprotein Mortality Risk (AMORIS) cohort^60^, and C-reactive protein, a marker of systemic inflammation, was reported to be associated with breast cancer via meta-analysis^61,62^. To our knowledge, no previous studies have reported the association of plasma phosphate, sodium in urine or creatinine in urine with breast cancer.

Our findings of novel predictors should be treated with caution, and further examined in independent datasets. These novel features could be surrogates for other processes that are not modelled in our analyses. The role of ML in this study is model-based feature selection that ranks the approximately 1.7k input features. The classical Cox model was our pre-specified model for risk prediction and statistical inference. Although we have pre-specified our analytical models, there is still the possibility of chance findings, particularly when the same training data were used in both the feature selection (i.e. the ML model) and the risk prediction (i.e. the classical Cox model). We did not observe evidence of reverse causation, but such absence of evidence should not be regarded as evidence of absence.

### Undiscovered well-established risk factors

Four well-established risk factors are unavailable (mammographic density, plasma oestrogen, progesterone) or unusable (plasma oestradiol) in the UKB. Plasma oestradiol was measured in UKB, but the measured concentrations of nearly all post-menopausal women were below the reportable range^13^, hence were regarded as missing values and could not be included in the analysis.

We identified 18 established risk factors for post-menopausal breast cancer from the literature, 14 of which are available in UKB. Out of these 14 risk factors, 4 were not ranked in the top 20 by the SHAP values: BMI was ranked at 79, probably because its related factor (e.g. basal metabolic rate) were already ranked among the top 20; HRT use was on the borderline of inclusion in top 20 (ranked 26), whereas age at menarche and parity were ranked further behind (ranked 74 and 72 respectively).

This highlights the need for establishing a criterion to decide which features should be regarded as important. Some suggested considering 3% of total number of features as important^63^, while others advocated a cut-off value of 0.05 for the SHAP feature importance measure^16^. Different approaches, such as Boruta^64^, exist, but all contain an element of randomness and there is no gold standard threshold. We pre-specified the top 20 features due to the practical need for keeping the number of features in a manageable range, in the absence of established criteria on this empirical choice. Apart from the SHAP values, importance can be similarly quantified in terms of gain in prediction power or even beyond the context of the model itself (e.g. cost-effectiveness of the model).

### PRS

We had an a priori interest in PRS, and intended to explore the relationship between the two PRS for breast cancer based on the existing understanding of their correlation. We discovered that both PRS were ranked as the strongest risk factors by the agnostic ML methods, which is surprising given that both PRS were developed using largely the same GWAS data for the same disease. We then conducted in-depth Cox regression simultaneously fitting both PRS as predictors, and concluded that both PRS are significant predictors for post-menopausal breast cancer. This raises the general question of whether multiple PRS should be used to improve risk prediction obtained from a single PRS.

SHAP dependence plots are useful for (i) visualising non-linear relationships between features and outcome, and (ii) revealing potential pairwise interactions between features. However, in this study, we noticed unexpected patterns arising from these plots, indicating that careful investigations are required before drawing firm conclusions.

### From ML to classical statistical models

ML methods are well suited for model-based large-scale (e.g. thousands) feature selection, and our SHAP values utilise the impact in model predictions with and without a particular feature to aid interpretability. In contrast, classical (e.g. backwards) feature selection methods are more suitable for selecting from a small number (e.g. 10–20) of features. Multivariable Cox models are beneficial in the subsequent risk prediction step, because (i) they are able to quantify the strength of association of each feature with the outcome via hazard ratio with 95% confidence interval; (ii) model coefficients (i.e. betas) and confidence intervals are typically used to infer feature importance in classical statistical models, providing a straightforward interpretation of the model output; and (iii) different choices of loss-function or feature ranking method in ML may result in different features being selected. It is therefore desirable to further examine the selected features using classical models for risk prediction.

When the search scope includes highly correlated features, one needs to carefully choose ML (e.g. tree-based) models that are capable of handling correlations, and then perform correlation and collinearity checks before including the selected features in classical statistical models. To reduce collinearity, we eliminate one feature among the highly correlated pairs, followed by checking the VIF. Other approaches for handling collinearity include Ridge and Lasso regression. It is possible that the eliminated feature could be a causal factor behind the retained feature. Furthermore, the focus of this paper is association (not causation), and therefore factors appearing to be strongly associated with the outcome do not necessarily imply causal relationships.

We did not expect the results obtained from a ML method to be fully compatible with those from a classical statistical model, because each approach has its own strengths and limitations. Instead, we aimed to complement Cox models with the insight from the XGBoost machine in this study. There are several possible explanations on the observed differences between the ML method and classical Cox models in this study.

First, our XGBoost machine characteristically makes binary splits of the input features among thousands of trees assuming non-linear relationship among features, whereas our Cox models are essentially linear. Although it is possible to incorporate non-linearity in Cox models (e.g. using splines or fractional polynomials), it would result in an overly complex model for interpretation. It is neither necessary nor appropriate to anticipate full agreement between ML and classical statistical models. Second, the criteria for inferring feature importance are different between ML and classical models, as described above.

Finally, we emphasise that while agreement between ML and classical models raises the confidence of discovery, differences do not necessarily imply superiority of either approach or compel us to choose one over the other. The differences could serve as a signal for further investigations where critical thinking should be exercised.

### Challenges and solutions

It is worth highlighting the challenges we encountered when implementing our analysis pipeline, and providing potential solutions here. The main challenge is how to handle missing data when combining ML and classical statistics for interpretation. Existing literature^65,66^ has compared various imputation strategies (e.g. response augmentation framework, K-nearest neighbour, mean imputation) and suggested utilising the method that yields the best prediction accuracy depending on the dataset. However, the potential biases arising from missing data must not be overlooked, particularly in the context of statistical modelling.

It is difficult to keep a consistent approach for handling missing data between ML and classical statistics. In classical statistics, one usually performs multiple imputation that generates multiple completed datasets (usually around 10–20), fits the model using each dataset, and then pools model estimates using Rubin’s rule^67^. However, it is not feasible to train a ML model on each imputed dataset due to the extensive computation time this would require. One alternative is to perform a single imputation, but this was not compatible with our complex variables where some are missing at random and some are not. For example, “Age at first birth” is not missing at random and if one blindly performs a single imputation, the imputed data would not make sense for women who have not had children. Although manual inspections can be performed on small datasets, it is practically impossible to carefully assess the missing mechanisms of the high ($\approx$ 1.7 k) dimensional data in our study.

Our solution is as follows: For the ML analysis, we treated missing data as a separate category and used the default setting for XGBoost (i.e. when the value needed for the split was missing, a default direction was assigned with the maximum gain). Once we had reduced the number of features to a workable size ($\approx$ 20), we carefully inspected the missingness and performed multiple imputation as appropriate in the classical statistical setting. One might argue that the missing mechanism for each feature should be consistent throughout the analyses, but such purity is impractical when dealing with large datasets. This issue could be a potential topic for future research.

### Strengths and limitations

We performed an agnostic search of potential predictors for breast cancer in post-menopausal women among $\approx$ 1.7 k features. We utilised an analysis pipeline for combined ML and classical statistical models, and incorporated necessary statistical considerations in our pipeline while acknowledging the anticipated inconsistency between different models. The presence of well-established risk factors, the large sample size, and the long follow-up period of UKB data have enabled us to perform rigorous analyses in the process.

Our study has several limitations. A few well-known risk factors (e.g. mammographic density, plasma oestrogen, progesterone, plasma oestradiol) and detailed family history data were either unavailable or unusable in UKB, hence could not be investigated in our study. Cohort-wide exposure data was captured at a single time point (date of enrolment), therefore we were unable to account for within-person variability for the input features. This means that our analysis pipeline was not suitable to incorporate longitudinal data of input features. We did not investigate subtypes (Estrogen-receptor [ER]-positive or negative) of breast cancer, due to incomplete data on tumour type in UKB. We did not incorporate exome data that are necessary for identifying BRCA1/BRCA2 carriers, or other high penetrance variants. Our study population consists only of genetically White individuals, and therefore should be not generalised to other ethnicities without further research. Moreover, our findings were solely based on UKB. Such recruitment-based biobanks are subject to potential biases, including “healthy volunteer” selection bias^68^ and informative missingness. External cohorts with different population characteristics are needed to further validate our findings. Finally, we did not compare our model with existing risk prediction models for breast cancer, such as BOADICEA^28^, Cuzick model^29^, due to their complexity and incomplete mapping to UKB variables.

### Conclusions

In conclusion, using ML for feature selection prior to risk prediction by Cox models, we identified five statistically significant novel associations with post-menopausal breast cancer for blood counts, blood biochemistry and urine biomarkers. The discrimination performance was maintained when adding these five novel features to the baseline Cox model that only contains two PRS and established risk factors. Adding such factors to a model does not substantively increase risk prediction, but, when included, appears to be relied upon when making subsequent risk predictions (as shown by the statistically-significant magnitude of model coefficients for these associated factors). We discovered that both of our pre-specified PRS were ranked as the most important features by SHAP value, and can be simultaneously included in our final Cox model. Our findings support further investigation on using more precise anthropometry measures for improved breast cancer prediction. The important next step would be to externally validate our findings, which is necessary before any subsequent implementation in clinical practice. Specific directions for future research include the utility of multiple PRS for better risk prediction, and validating the association between blood/urine biomarkers and risk of breast cancer using data external to UKB.

## Supplementary Information

Supplementary Information 1.

Supplementary Information 2.

## Data Availability

The data reported in this paper are available via application directly to the UK Biobank, https://www.ukbiobank.ac.uk. The corresponding preprint is available on medRxiv 2022.06.27.22276932. The code used for analyses are available at https://github.com/xiaonanl1996/MLforBrCa. Section 7 in supplementary material provides more descriptions on code structure and analysis workflow.
