# Supplementary material for: Combining machine learning with Cox models to identify predictors for incident post-menopausal breast cancer in the UK Biobank
Source: Sci Rep. 2023 Jun 7;13:9221. doi: 10.1038/s41598-023-36214-0 (PMC10247810; doi:10.1038/s41598-023-36214-0)
Supplement: Supplementary file 1 — Supplementary Information 1. [file 41598_2023_36214_MOESM1_ESM.docx]

Combining machine learning with Cox models to identify predictors for incident post-menopausal breast cancer in the UK Biobank

(Supplementary Materials)

Xiaonan Liu^1,3*^, Davide Morelli^2^, Thomas J Littlejohns^1,3^, David A Clifton^2^, Lei Clifton^1,3^

Affiliate Institutions:

1. Nuffield Department of Population Health, University of Oxford, Oxford, UK.

2. Department of Engineering Science, University of Oxford, Oxford, UK.

3. Big Data Institute, Old road campus, University of Oxford, Oxford, OX3 7LF, UK.

*Corresponding Author: Xiaonan Liu

Mailing address: Big Data Institute, Old road campus, University of Oxford, OX3 7LF.

Email: [xiaonan.liu@ndph.ox.ac.uk](mailto:xiaonan.liu@ndph.ox.ac.uk)

Tel: [01865 287770](https://www.google.com/search?gs_ssp=eJzj4tVP1zc0TDZLziivKCoxYLRSNagwsTA3SzY0M002STVLNTIztTKosDRPSTY1SjY2MEu0TDM3TPESSspMV0hJLElUyMwrLsksKS1JBQAWvRZV&q=big+data+institute&rlz=1C1CHBF_enGB909GB909&oq=big+data+&aqs=chrome.1.69i57j46i39i175i199j0i67l2j69i60l2j69i65j69i60.3940j0j7&sourceid=chrome&ie=UTF-8)

ORCID: 0000-0002-2930-605X

# Genetic QC

The UK Biobank (UKB) genetic data were assayed using two closely related arrays where ~49k participants were genotyped using the UK BiLEVE array and ~438k participants were genotyped using the UKB Axiom array. UKB computed the top 40 principal components using a subset of high quality, unrelated samples to measure population structure within UKB.

Supplementary Table 1. Number of SNPs excluded during SNP quality control (QC) of PRS for breast cancer. PRS: Polygenic risk scores. nSNPs: Number of single nucleotide polymorphisms (SNPs) included in the PRS prior to SNP QC. UKB: UK Biobank.

| PRS | nSNPs | Unavailable in UKB | Ambiguous | Imputation info < 0.4 | MAF < 0.005 | Remaining SNPs |
| --- | --- | --- | --- | --- | --- | --- |
| PRS_313_ | 313 | 7 | 0 | 0 | 1 | 305 |
| PRS_120k_ | 118,388 | 0 | 43 | 107 | 2938 | 115,300 |

# Input features

Supplementary Table 2. Input features to the machine learning (ML) model for risk factor discovery of post-menopausal breast cancer (BrCa). The “Category” column specifies variables at the "level 2" category defined by UK Biobank (UKB) (see [UKB website](https://biobank.ndph.ox.ac.uk/showcase/browse.cgi)). The “Sub-category” column specifies variables at the "level 3" category by UKB. The “Variables” column lists the variables selected for consideration with justification in the “Comments” column. FID: Field ID in UKB. Symbol "--" stands for "not applicable" or "not included". Pre-processing on missing categories (i.e. “Prefer not to answer”, “Do not know” and empty entry) were applied to all variables below.

| Category | Sub-category | Variables | Comments |
| --- | --- | --- | --- |
| PRS | -- | PRS_313_ and PRS_120k_ | We computed PRS using imputed genetic data from UKB (version 3, March 2018 release) via pipeline ^1^. Full details in the manuscript. |
| [Socio-demographics category](https://biobank.ndph.ox.ac.uk/showcase/label.cgi?id=701) | [Baseline characteristics](https://biobank.ndph.ox.ac.uk/showcase/label.cgi?id=100094) | Age at baseline, Townsend deprivation score | We did not include gender and ethnicity in the input features, because our study population contains only genetically White females.  We combined FID 6138 and 10722 into one variable called “Highest qualifications achieved”. |
|  | [Education](https://biobank.ndph.ox.ac.uk/showcase/label.cgi?id=100063) | Qualifications |  |
|  | [Employment](https://biobank.ndph.ox.ac.uk/showcase/label.cgi?id=100073) | Employment status, [employment category](https://biobank.ndph.ox.ac.uk/showcase/field.cgi?id=20277) |  |
|  | [Household](https://biobank.ndph.ox.ac.uk/showcase/label.cgi?id=100066) | Household (pre-tax) income |  |
|  | [Indices of Multiple deprivation](https://biobank.ndph.ox.ac.uk/showcase/label.cgi?id=76) | -- |  |
| [Lifestyle category](https://biobank.ndph.ox.ac.uk/showcase/label.cgi?id=704) | [Alcohol](https://biobank.ndph.ox.ac.uk/showcase/label.cgi?id=100051) | Alcohol drinking status, weekly alcohol consumed units | We derived weekly alcohol consumed units by combining consumption from red wine, white wine, beer, cider, spirits, fortified wine and other alcohol. |
|  | [Diet](https://biobank.ndph.ox.ac.uk/showcase/label.cgi?id=100052) | Variables within whole category | Some variables (e.g. [FID 1309](https://biobank.ndph.ox.ac.uk/showcase/field.cgi?id=1309): Fresh fruit intake) contain special values (e.g. “-10” represents “Less than one”), which were coded as 0. |
|  | [Electronic device use](https://biobank.ndph.ox.ac.uk/showcase/label.cgi?id=100053) | Weekly usage of mobile phone in last 3 months | We excluded pilot field [FID 10749](https://biobank.ndph.ox.ac.uk/showcase/field.cgi?id=10749) because its categories do not match with main field (FID 1120). |
|  | [Physical activity](https://biobank.ndph.ox.ac.uk/showcase/label.cgi?id=100054) | Variables within whole category | All pilot fields were omitted because their categories are different from those of the main fields, which made them impossible to be combined. |
|  | [Sleep](https://biobank.ndph.ox.ac.uk/showcase/label.cgi?id=100057) | Variables within whole category | These include sleep duration, getting up in morning, morning/evening person, napping during day, insomnia, snoring, daytime dozing/sleeping. |
|  | [Smoking](https://biobank.ndph.ox.ac.uk/showcase/label.cgi?id=100058) | Smoking status | We used raw UKB variable. |
|  | [Social support](https://biobank.ndph.ox.ac.uk/showcase/label.cgi?id=100061) | Leisure/social activities  Able to confide, frequency of friend/family visits | We used raw UKB variables. |
|  | [Mental health](https://biobank.ndph.ox.ac.uk/showcase/label.cgi?id=100060) | -- | We did not use this self-report category, and instead used ICD-10 codes from “Chapter V Mental and behavioural disorders”. |
|  | [Sun exposure](https://biobank.ndph.ox.ac.uk/showcase/label.cgi?id=100055) | Variables within whole category | We used raw UKB variables. |
| [Family History](https://biobank.ndph.ox.ac.uk/showcase/label.cgi?id=705) | -- | Family history (FaH) of CVD (high BP, stroke, heart disease), FaH of breast cancer, FaH of severe depression, FaH of diabetes, FaH of other cancer (bowel cancer, prostate cancer, lung cancer), FaH of dementia, FaH of Parkinson’s disease, FaH of chronic bronchitis  Variables within rest of the whole category (except fields related to adopted family members) | FaH variables were derived from [illness of father](https://biobank.ndph.ox.ac.uk/showcase/field.cgi?id=20107), [mother](https://biobank.ndph.ox.ac.uk/showcase/field.cgi?id=20110), [siblings](https://biobank.ndph.ox.ac.uk/showcase/field.cgi?id=20111).  We did not incorporate variables regarding adopted father, mother and siblings. |
| [Early life and reproductive factors](https://biobank.ndph.ox.ac.uk/showcase/label.cgi?id=708) | -- | Variables within [Female-specific factors](https://biobank.ndph.ox.ac.uk/showcase/label.cgi?id=100069) and [Early Life factors](https://biobank.ndph.ox.ac.uk/showcase/label.cgi?id=100033) | Male-specific factors were not considered.  Female-specific variables that contain special values were coded as follows:  Value "-10" in variable FID 2704: “[Years since last cervical smear test](https://biobank.ndph.ox.ac.uk/showcase/field.cgi?id=2704)” represents less than a year ago, which was treated as 0.  Value "-6" in variable FID 3710: “[Length of menstrual cycle](https://biobank.ndph.ox.ac.uk/showcase/field.cgi?id=3710)” represents irregular cycle, which was treated as missing.  Value "-4" in variable FID 2764: “[Age at last live birth](https://biobank.ndph.ox.ac.uk/showcase/field.cgi?id=2764)” represents "Do not remember", which was treated as missing. |
| [Health conditions](https://biobank.ndph.ox.ac.uk/showcase/label.cgi?id=100091) | -- | Indicators of each [level 2 of Diagnoses ICD 10](https://biobank.ndph.ox.ac.uk/showcase/field.cgi?id=41270) (except unspecific categories (“Chapter XX”, “Chapter XXI”, “Chapter XXII”) | When deriving health conditions in UKB, one usually investigates multiple resources (e.g. ICD-10, ICD-9, self-report), and then defines the condition across them. However, in this study, we do not have pre-specified health conditions, and it is impractical to combine multiple resources for the definition of each of the 19k conditions present in UKB. Therefore, we utilised one dominant field, [hospital inpatient ICD-10 code](https://biobank.ndph.ox.ac.uk/showcase/field.cgi?id=41270), for medical condition diagnoses at baseline. |
| [Genotype results, process and QC](https://biobank.ndph.ox.ac.uk/showcase/label.cgi?id=716) | -- | -- | We used this category for computing PRS but fields within this category were not considered by the ML model. |
| [Procedural metrics](https://biobank.ndph.ox.ac.uk/showcase/label.cgi?id=718) | -- | -- | This category consists of admin variables, hence not included as input features. |
| [Medication](https://biobank.ndph.ox.ac.uk/showcase/field.cgi?id=20003) | -- | Self-report medication use within each ATC group at baseline from [Treatment/medication code](https://biobank.ndph.ox.ac.uk/showcase/field.cgi?id=20003) | We included usage of different medications in feature selection stage. Further details in the manuscript. |
| [Blood Assays](https://biobank.ndph.ox.ac.uk/showcase/label.cgi?id=100080) |  | [Blood biochemistry](https://biobank.ndph.ox.ac.uk/showcase/label.cgi?id=17518), [Blood counts](https://biobank.ndph.ox.ac.uk/showcase/label.cgi?id=100081) | Variable “[Rheumatoid factor](https://biobank.ndph.ox.ac.uk/showcase/field.cgi?id=30820)”, variable “[Oestradiol](https://biobank.ndph.ox.ac.uk/showcase/field.cgi?id=30800)”, all variables in category “[Infectious disease](https://biobank.ndph.ox.ac.uk/showcase/label.cgi?id=51428)”, all variables in category “[Metabolics](https://biobank.ndph.ox.ac.uk/showcase/label.cgi?id=220)” were not considered due to extremely high missing data.  (“Rheumatoid factor” was available for 8% of participants, “Oestradiol” was available for 15% of participants, and “Infectious disease” was available for 1.8% of participants and “Metabolics” was available for 3.5% of participants.) |
| [Urine Assays](https://biobank.ndph.ox.ac.uk/showcase/label.cgi?id=100083) | -- | Variables within whole category | We used raw UKB variables. |
| [Physical Measures](https://biobank.ndph.ox.ac.uk/showcase/label.cgi?id=100006) |  | Variables within [Blood pressure](https://biobank.ndph.ox.ac.uk/showcase/label.cgi?id=100011), [Hearing test](https://biobank.ndph.ox.ac.uk/showcase/label.cgi?id=100049), [Arterial stiffness](https://biobank.ndph.ox.ac.uk/showcase/label.cgi?id=100007), [Hand grip strength](https://biobank.ndph.ox.ac.uk/showcase/label.cgi?id=100019), [Anthropometry](https://biobank.ndph.ox.ac.uk/showcase/label.cgi?id=100008), [Eye measures](https://biobank.ndph.ox.ac.uk/showcase/label.cgi?id=100013), [Bone-densitometry of heel](https://biobank.ndph.ox.ac.uk/showcase/label.cgi?id=100018), [Spirometry](https://biobank.ndph.ox.ac.uk/showcase/label.cgi?id=100020) | Admin fields (e.g. Carotid ultrasound measurement completed) and bulk data fields (e.g. [ECG datasets](https://biobank.ndph.ox.ac.uk/showcase/field.cgi?id=20205)) were excluded from input features.  Two ECG categories were excluded from input features: (i) “[ECG at rest, 12-lead](https://biobank.ndph.ox.ac.uk/showcase/label.cgi?id=104)”, due to unavailability at baseline, and (ii) “[ECG during exercise](https://biobank.ndph.ox.ac.uk/showcase/label.cgi?id=100012)”, because its analysis requires expert knowledge.  For sub-categories of eye measures, only 2 fields within “[Visual acuity](https://biobank.ndph.ox.ac.uk/showcase/label.cgi?id=100017)” were considered because UKB website indicates that “*For non-specialists, the primary items of interest are*[*Field 5201*](https://biobank.ndph.ox.ac.uk/showcase/field.cgi?id=5201)*and*[*Field 5208*](https://biobank.ndph.ox.ac.uk/showcase/field.cgi?id=5208)*.*” “[Autorefraction](https://biobank.ndph.ox.ac.uk/showcase/label.cgi?id=100014)” was not considered because it requires expert knowledge to analyse. “[Retinal optical coherence tomography](https://biobank.ndph.ox.ac.uk/showcase/label.cgi?id=100016)” and “[Intraocular pressure](https://biobank.ndph.ox.ac.uk/showcase/label.cgi?id=100015)” only contain data for 100k participants, hence were not considered.  Within the “[Spirometry](https://biobank.ndph.ox.ac.uk/showcase/label.cgi?id=100020)” category, we only included FVC, FEV1 and PEF measures, by taking the average value of multiple measurements at baseline. |
| [Imaging](https://biobank.ndph.ox.ac.uk/showcase/label.cgi?id=100003) | -- | -- | Not available at baseline. |
| [Cognitive function](https://biobank.ndph.ox.ac.uk/showcase/label.cgi?id=100026) | [Reaction time](https://biobank.ndph.ox.ac.uk/showcase/label.cgi?id=100032) | [Mean time to correctly identify matches](https://biobank.ndph.ox.ac.uk/showcase/field.cgi?id=20023) | We used the raw UKB variable. |
|  | [Numeric memory](https://biobank.ndph.ox.ac.uk/showcase/label.cgi?id=100029) | [Maximum digits remembered correctly](https://biobank.ndph.ox.ac.uk/showcase/field.cgi?id=4282) | Only this field was considered because UKB website indicates that “*For non-specialists, the primary item of interest is*[*Field 4282*](https://biobank.ndph.ox.ac.uk/showcase/field.cgi?id=4282).” |
|  | [Fluid intelligence](https://biobank.ndph.ox.ac.uk/showcase/label.cgi?id=100027) | [Fluid intelligence score](https://biobank.ndph.ox.ac.uk/showcase/field.cgi?id=20016) | Only this field was considered because UKB website states “For non-specialists, the primary item of interest is [Field 20016](https://biobank.ndph.ox.ac.uk/showcase/field.cgi?id=4282).” |
|  | [Trial making](https://biobank.ndph.ox.ac.uk/showcase/label.cgi?id=505),  [Matrix pattern completion](https://biobank.ndph.ox.ac.uk/showcase/label.cgi?id=501),  [Tower rearranging](https://biobank.ndph.ox.ac.uk/showcase/label.cgi?id=503),  [Picture vocabulary](https://biobank.ndph.ox.ac.uk/showcase/label.cgi?id=504),  [Symbol digit substitution](https://biobank.ndph.ox.ac.uk/showcase/label.cgi?id=502),  [Paired associate learning](https://biobank.ndph.ox.ac.uk/showcase/label.cgi?id=506) | -- | Only available from imaging visit. |
|  | [Prospective memory](https://biobank.ndph.ox.ac.uk/showcase/label.cgi?id=100031) | [Prospective memory result](https://biobank.ndph.ox.ac.uk/showcase/field.cgi?id=20018) | We used the raw UKB variable. |
|  | [Pairs matching](https://biobank.ndph.ox.ac.uk/showcase/label.cgi?id=100030) | -- | UKB website states “*For non-specialists, the primary item of interest is*[*Field 399*](https://biobank.ndph.ox.ac.uk/showcase/field.cgi?id=4282) *and (for the pilot) FID 10137*.” However this primary field was unavailable. |
|  | [Lights pattern memory](https://biobank.ndph.ox.ac.uk/showcase/label.cgi?id=100028),  [Word production](https://biobank.ndph.ox.ac.uk/showcase/label.cgi?id=100077) | -- | Both categories contain only pilot fields and hence were not used. |

# Machine Learning (ML) Methods

## Hyper-parameter tuning of XGBoost

Parameters of the eXtreme Gradient Boosting (XGBoost) algorithm fall into two main categories: (i) tree-specific parameters that guide the structuring of individual trees at each step, and (ii) regularisation parameters that reduce the chance of over-fitting and enhance generalisation (full details in Supplementary Table 3).

The large set of tree-specific and regularisation parameters enables the construction of sophisticated models for better prediction, but adds difficulty in searching for the optimal parameters. Unlike classical statistical models whose parameters are directly estimated from the data, XGBoost machines usually require manual specification of parameters due to their complexity. Such parameters are often referred to as hyper-parameters, and the choice of their values has a direct impact on the model performance. Therefore, the process of searching for parameters that yield the optimal model (i.e. hyper-parameter tuning) is crucial for training an XGBoost machine.

Common strategies for hyper-parameter tuning include grid search ^23^, random search ^4^ , and Bayesian optimization ^5^. In this study, we applied grid search with 5-fold cross-validation (CV) on training data using negative partial log-likelihood for Cox proportional hazard model (i.e. cox-loss) as the evaluation metric.

During the grid search, each combination of parameters specified in a grid was fitted to the model. For example, if one specifies a grid with depth of tree set to be {2, 3, 4} and number of trees to be {10, 20}, the grid search will fit the model with each of 6 possible combinations. The optimal combination was obtained by computing the average cox-loss obtained from the five validation sets and selecting the set with the lowest value.

The drawback of this approach is its high computational cost for high-dimensional search spaces, which XGBoost is subject to due to its large set of parameters. Therefore, we further applied the following tuning strategy to improve the efficiency of the grid search.

We started with a relatively high learning rate of 0.1 to determine the corresponding approximate number of trees. We then tuned the tree-specific parameters followed by the tuning of regularisation parameters. Finally, we lowered the learning rate and increased the number of trees accordingly to obtain a more robust model (Supplementary Table 3).

Supplementary Table 3. Hyper-parameter tuning strategy of our XGBoost machine via grid search with 5-fold CV. We followed the naming convention of parameters by [Scikit-Learn API](https://xgboost.readthedocs.io/en/stable/python/python_api.html#module-xgboost.sklearn). min(Coxloss_CV_): Highest AUC obtained from 5-fold CV on training data.

| Steps | Search Ranges of parameters | Explanation |
| --- | --- | --- |
| Step 1: Build a baseline model | learning_rate$=$0.1 early_stopping_rounds$=$50 n_estimators$\in$ [1..500] | Learning rate (learning_rate) is inversely correlated with the number of trees (n_estimators). Lower learning rates typically yield better model performance, given a sufficient number of trees. However, computation becomes expensive with decreased learning rate and increased number of trees. Constructing a baseline model with a relatively high learning rate produces an approximate estimate of the number of trees required for a specific learning rate without sacrificing computational power.  Early stopping works as follows: for a pre-specified early stopping rounds (early_stopping_rounds) (e.g. 10), the model will train until there is no improvement in validation score in the next 10 rounds. This enables users to grow a complex model, minimise the chance of over-fitting, save computation time, and obtain a more accurate number of trees. |
| Step 2: Tune max_depth and min_child_weight | max_depth $\in$ [1..4]  min_child_weight$\in$ {1, 3, 5}  min(Coxloss_CV_)$=$9.449 | Parameter max_depth specifies the maximum depth of tree. The higher the value, the more complex the model. Parameter min_child_weight defines the minimum sum of weights of all observations required in a child. The higher the value, the more conservative the model. |
| Step 3: Tune gamma | gamma $\in$ {0, 0.2, 0.4, 0.6, 0.8, 1}  min(Coxloss_CV_)$=$9.449 | Parameter gamma is the minimum loss reduction required to make a split. The higher the value, the more conservative the model. |
| Step 4: Tune subsample and colsample_bytree | subsample$\in$ {0.6, 0.7, 0.8, 0.9}  colsample_bytree$\in${0.6,0.7,0.8,0.9}  min(Coxloss_CV_)$=$9.449 | Parameter subsample is the fraction of observations to be randomly sampled for each tree. Parameter colsample_bytree is the fraction of features to be randomly sampled for each tree. |
| Step 5: Tune regularisation parameters | lambda $\in$ {8, 10, 12, 14, 16, 18, 20, 22}  min(Coxloss_CV_)$=$9.447 | For regularisation parameters lambda and alpha, one has the option to choose which one to use for penalising weights of leaves: lambda corresponds to L2 regularisation term that encourages weights to be small, whereas alpha corresponds to L1 regularization that encourages weights to be 0. Here we took a more conservative approach for tuning lambda instead of alpha. |
| Step 6: Lower **learning_rate** and increase **n_estimators** | learning_rate $\in$ {0.001, 0.003, 0.005, 0.007, 0.01}  n_estimators$\in$[1..50000]  early_stopping_rounds=200  min(Coxloss_CV_)$=$9.442 | Here we lowered the learning rate (learning_rate) and increased the number of trees (n_estimators) for a more robust model. |


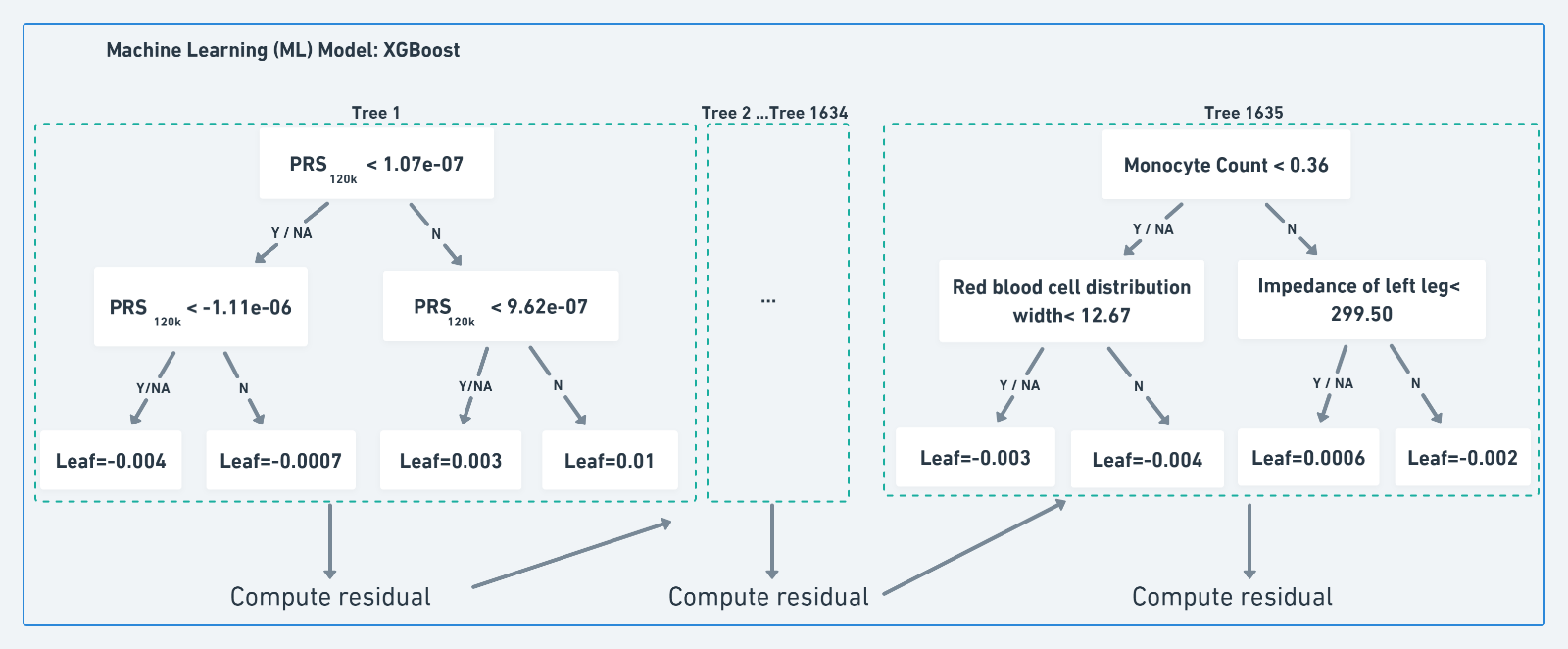


*Supplementary Figure 1. The structure of the optimal XGBoost model, trained using cox-loss. For illustrative purpose, only the structures of the first and last trees are outlined. Y/NA: Yes/Missing. N: No. Monocyte count, 10^9 cells/Litre. Red blood cell distribution width, percent. Impedance of left leg, ohms.*

## SHAP values

SHapley Additive exPlanation (SHAP) values originated from game theory ^6^ as a metric to compute fair contribution among players. It has been recently incorporated into machine learning models where it is being developed to aid model interpretation. For the purpose of feature selection, SHAP values compute the marginal contribution of each feature to the model prediction among all possible coalitions, which takes account of interactions among features.

As a member of the additive feature attribution methods, it uses a simple explanation model to approximate the prediction from a complex machine learning model ^7^:

|  | $f\left( \boldsymbol{x} \right)=g\left( \boldsymbol{x}^{'} \right)= \emptyset_{0}+ \sum_{i=1}^{M} \emptyset_{i}\boldsymbol{x}_{i}^{'}$ | (1) |
| --- | --- | --- |

Where $\boldsymbol{x}$ is the original input feature, $\boldsymbol{x}^{\boldsymbol{'}}$ is the simplified input, mapped through a function where **﻿**$\boldsymbol{x}=h_{x}(\boldsymbol{x}^{\boldsymbol{'}})$, $f\left( \boldsymbol{x} \right)$ is the model output that is computed by default in log odds scale for binary classifications, $\emptyset_{0}$ is the output when no input is present, *M* is the total number of features, and $\emptyset_{i}\mathbb{\in R}$ is the attribution value (i.e. SHAP value) of feature $i$ ^7^.

The SHAP value of feature $i$ is computed as follows: for each possible subset of features $S\subseteq F\backslash\{i\}$ (where $F$ represents the set of all features), two models are trained incorporating the potential dependency between features: one including feature $i$ and one without it. The difference between the outputs of the two models is then computed as $f_{S\cup\{i\}}\left( \boldsymbol{x}_{S\cup\{i\}} \right)- f_{S}\left( \boldsymbol{x}_{S} \right)$, where $\boldsymbol{x}_{S}$ indicates the values of input features in the subset $S$. $\emptyset_{i}$ is computed as the weighted average of all possible differences, where the weight captures the feature space of each subset, shown in Equation (2),

|  | $\emptyset_{i}= \sum_{S\subseteq F\backslash\{i\}} \frac{\left\vert S \right\vert!\left( M-\left\vert S \right\vert-1 \right)!}{M!} \left[ f_{S\cup\{i\}}\left( \boldsymbol{x}_{S\cup\{i\}} \right)- f_{S}\left( \boldsymbol{x}_{S} \right) \right]$ | (2) |
| --- | --- | --- |

﻿where $F$ is the set of all features. $\left| S \right|!$ is the number of features in $S$ where $S\subseteq F\backslash\{i\}$ represents all the possible subsets without feature $i$.

A major challenge for computing SHAP values is that the computation cost grows exponentially as the number of features increases. This is resolved by computing SHAP values locally (i.e. each feature attribution is computed using one sample) ^8^. For tree-based ML models, the internal tree structure is utilised for faster computation.

More specifically, for a dataset with $N$ samples and $M$ features (i.e. $N\times M$), SHAP values are generated in the same dimension where the attribution of feature $i$, $\emptyset_{ij}$, is computed locally using each sample for individual $j$. In the summary bar plot (e.g. Supplementary Figure 1), the mean absolute SHAP (SHAP_ma_) value of each feature is aggregated by taking the mean over all samples:

$${\mathrm{SHAP}\mathrm{ma}}_{i}= \frac{1}{N} \sum_{j=1}^{N} |\emptyset_{ij}|$$

In addition to SHAP values, we also implemented two different feature importance methods: XGBoost default feature importance (“weight”) and permutation based feature importance. We observed inconsistency between the XGBoost default feature importance (“weight”) ranking and the SHAP values ranking. The permutation based method required high computation cost compared with the others, and due to high collinearity between features it did not produce feasible results for further inspection.

## XGBoost with log-loss

In the current manuscript, we used survival outcomes of breast cancer to train XGBoost to minimise the cox-loss, as suggested by the reviewer. Originally, we used binary indicator of breast cancer status to implement XGBoost with log-loss. For hyper-parameter tuning, we performed grid search with five-fold CV on training data using Area Under the receiver operating characteristic Curve (AUC) as the evaluation metric. The optimal set of hyper-parameters for XGBoost were found to be: maximum depth $=$ 2, number of trees $=$ 1,571, learning rate $=$ 0.01, minimum of child weights $=$ 3, gamma $=$ 0.8, subsample $=$ 0.8, column sample by tree $=$ 0.9, lambda for regularisation $=$ 18, and scale positive weight $=$ 1. The AUC obtained from the five-fold CV was 0.6680 on training data and 0.6679 on test data, indicating that the model was not over-fitted. The top 20 features with SHAP values from XGBoost trained using log-loss are shown in Supplementary Figure 2.


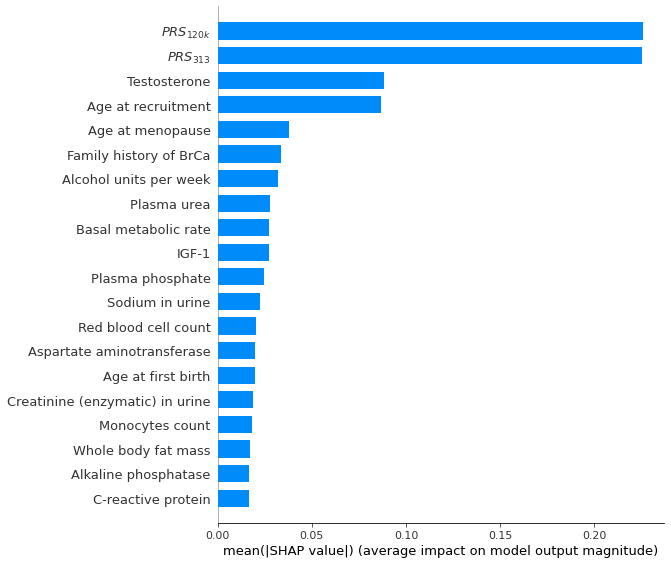


Supplementary Figure 2. SHAP summary bar plot showing the top 20 most important features for the risk of breast cancer, according to the XGBoost machine (trained using **log-loss**) and SHAP values. BrCa: Breast Cancer. SHAP: SHapley Additive explanation. mean(|SHAP value|): mean absolute SHAP value, SHAP_ma_.

## HistGBM

As a sensitivity analysis, we further investigated the robustness of feature ranking by exploring another ML method, the Histogram-based Gradient Boost Machines (HistGBM), inspired by LightGBM ^9^. Traditional GBM and HistGBM select the split points differently during the construction of trees. Traditional GBM sorts values of each continuous feature, and considers the average of each pair of adjacent values as splitting points when building trees, which can be computationally expensive for high-dimensional data. HistGBM improves the computational efficiency by discretising (i.e. binning) the continuous features to construct feature histograms (255 bins by default) during training, which significantly decreases the number of splitting points. This approximation often has little impact on model performance but dramatically reduces the memory consumption and accelerates the training speed. Besides faster implementation for large datasets, HistGBM also handles missing data by default (in the same way as XGBoost) and utilises L2 regularization to reduce over-fitting.

We adapted the same hyper-parameter tuning process (grid search with 5-fold CV) as in our XGBoost machine, except that HistGBM has fewer parameters to tune (Supplementary Table 4).

Supplementary Table 4. Hyper-parameter tuning strategy of HistGBM via grid search with CV. We followed the naming convention of parameters by [Scikit-Learn API](https://scikit-learn.org/stable/modules/generated/sklearn.ensemble.HistGradientBoostingClassifier.html). max(AUC_CV_): Highest AUC obtained from 5-fold CV on training data.

| Steps | Search Ranges of parameters |
| --- | --- |
| Step 1: Build a baseline model | learning_rate$=$0.1  n_iter_no_change*$=$50  max_iter* $\in$[1..500] |
| Step 2: Tune tree-specific parameters | max_dpeth $\in$ [1..8]  min_samples_leaf* $\in${20, 30,40,50,60,70}  max(AUC_CV_)$=$ 0.663 |
| Step 3: Tune L2 regularisation parameter | lambda $\in${1e-6, 1e-5, 5e-5, 0, 0.01, 0, 0.1, 1, 5, 10, 20, ,25, 30, 35, 40, 45, 50, 100}  max(AUC_CV_)$=$0.664 |
| Step 4: Lower **learning_rate** and increase **max_iter** | learning_rate $\in$ {0.1, 0.01, 0.001}  n_estimators$\in$[1..50000]  early_stopping_rounds$=$200  max(AUC_CV_)$=$0.664 |

*Note*:* n_iter_no_change *controls when to early stop (analogous to* early_stopping_rounds *in XGBoost);* max_iter *represents the number of trees;* min_samples_leaf *defines minimum samples per leaf.*

During Step 4 of hyper-parameter tuning, we observed the model with learning_rate$=$0.001 had the highest AUC; however, the improvement compared to model with learning_rate$=$0.01 was modest. After taking computation time into account, the optimal set of hyper-parameters for HistGBM were found to be: learning_rate $=$ 0.01, max_iter $=$ 1,366, max_depth $=$ 2, min_samples_leaf $=$ 30, lambda $=$ 35. The AUC obtained from the 5-fold CV on training data was 0.664 and the AUC on test data was 0.663.

The top 20 features with SHAP values from HistGBM are shown in Supplementary Figure 3. We observed a high level of overlap with those from XGBoost trained with log-loss (Supplementary Figure 2), which confirms the robustness of our risk factor discovery.

The six new features not selected by XGBoost are:

- Physical activity features (Duration walking for pleasure, Summed MET minutes per week for all activity)
- Hormone (SHBG)
- Anthropometry (Trunk fat-free mass)
- Others (Country of birth (UK/elsewhere), UK country of residence).

Three of these six were on the borderline of being included in the top 20 by XGBoost (trained using log-loss) and SHAP values; they are: trunk fat-free mass (ranked 21), summed MET minutes per week for all activity (ranked 23), and SHBG (ranked 29).

Although duration of walking for pleasure was ranked 10 by HistGBM (but only 135 by XGBoost), it may have relatively little impact since the more detailed physical activity feature, summed MET minutes per week for all activity, was already ranked among the top 20 features by HistGBM. Variables in the "Others" category could be due to the artefacts of censoring, e.g. the short follow-up time in Wales may have contributed to fewer breast cancer cases there.


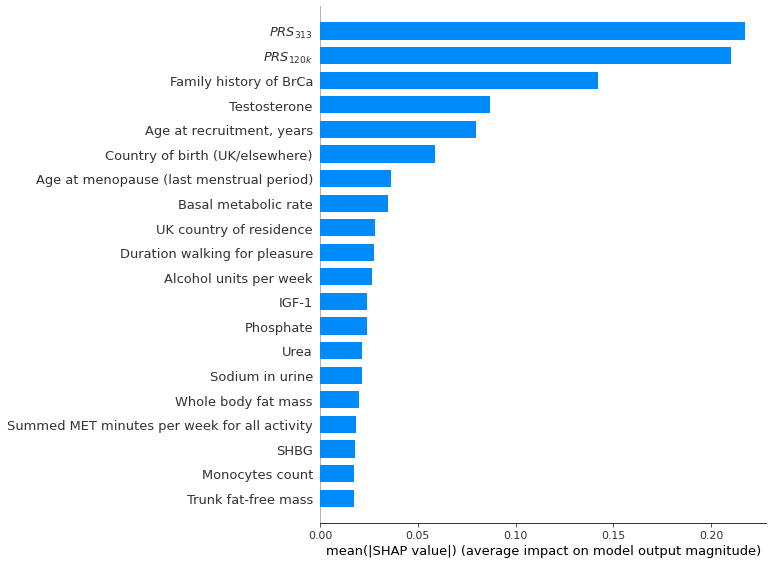


Supplementary Figure 3. The top 20 most important features for the risk of breast cancer (BrCa), according to the HistGBM and SHAP values. Noticeably, both BrCa PRS are deemed of much higher importance than the remaining phenotypic features. BrCa: Breast Cancer. SHAP: SHapley Additive explanation. mean(|SHAP value|): Mean absolute SHAP value.

# Tables

## Correlation matrix

Supplementary Table 5. Pairs of features whose correlation coefficient is above 0.5, among the union of top 20 features and other established predictors using the training data.

| Features | Features | Correlation |
| --- | --- | --- |
| BMI | Basal metabolic rate | 0.73 |
| $PRS_{120k}$ | $PRS_{313}$ | 0.63 |
| Creatinine (enzymatic) in urine | Sodium in urine | 0.54 |

## Full Cox model output

Supplementary Table 6. Cox regression of time until breast cancer in post-menopausal women (3,252 events) run in training data. Baseline model represents multivariable Cox model containing two PRS and established risk factors. New model represents multivariable Cox model containing all covariates in baseline model and novel features discovered by XGBoost.

|  | Baseline model | | | New model | | |
| --- | --- | --- | --- | --- | --- | --- |
| Coefficient | HR | 95% CI | p | HR | 95% CI | p |
| $PRS_{120k}$ | 1.34 | (1.28, 1.40) | <0.001 | 1.33 | (1.27, 1.39) | <0.001 |
| $PRS_{313}$ | 1.32 | (1.26, 1.38) | <0.001 | 1.32 | (1.26, 1.38) | <0.001 |
| **Age at recruitment, years** | 1.03 | (1.02, 1.04) | <0.001 | 1.04 | (1.03, 1.04) | <0.001 |
| **BMI, kg/m^2** | 1.03 | (1.02, 1.03) | <0.001 | 1.00 | (0.98, 1.01) | 0.453 |
| **Age at menopause, years** | 1.02 | (1.01, 1.02) | <0.001 | 1.01 | (1.01, 1.02) | <0.001 |
| **Daily alcohol intake, units** | 1.07 | (1.04, 1.09) | <0.001 | 1.06 | (1.04, 1.09) | <0.001 |
| **Age at first birth, years (Categorical)** |  |  |  |  |  |  |
| No Births | 1 |  |  | 1 |  |  |
| <20 | 1.04 | (0.85, 1.27) | 0.703 | 1.09 | (0.89, 1.33) | 0.418 |
| 20-30 | 1.06 | (0.92, 1.23) | 0.398 | 1.10 | (0.95, 1.27) | 0.218 |
| 30-40 | 1.23 | (1.06, 1.43) | 0.006 | 1.24 | (1.07, 1.44) | 0.004 |
| >=40 | 1.09 | (0.71, 1.66) | 0.696 | 1.09 | (0.71, 1.66) | 0.700 |
| **Family history of BrCa** |  |  |  |  |  |  |
| No family history of BrCa | 1 |  |  | 1 |  |  |
| Family history of BrCa | 1.32 | (1.20, 1.45) | <0.001 | 1.31 | (1.19, 1.44) | <0.001 |
| **Summed MET minutes per week** | 0.95 | (0.91, 1.00) | 0.033 | 0.95 | (0.91, 1.00) | 0.035 |
| **HRT user** | 1.89 | (1.58, 2.26) | <0.001 | 1.68 | (1.40, 2.03) | <0.001 |
| **Age at menarche, years** | 0.99 | (0.96, 1.01) | 0.208 | 0.98 | (0.96, 1.00) | 0.103 |
| **IGF-1, nmol/L** | 1.01 | (1.00, 1.02) | <0.001 | 1.01 | (1.01, 1.02) | <0.001 |
| **Testosterone, nmol/L** | 1.12 | (1.08, 1.16) | <0.001 | 1.11 | (1.07, 1.16) | <0.001 |
| **Number of live births** | 0.94 | (0.90, 0.99) | 0.018 | 0.94 | (0.89, 0.98) | 0.008 |
| **Plasma urea, mmol/L** |  |  |  | 0.95 | (0.92, 0.98) | <0.001 |
| **Basal metabolic rate, KJ** |  |  |  | 1.17 | (1.11, 1.24) | <0.001 |
| **Aspartate aminotransferase, U/L** |  |  |  | 1.00 | (0.99, 1.00) | 0.115 |
| **Plasma phosphate, mmol/L** |  |  |  | 0.68 | (0.53, 0.88) | 0.003 |
| **C-reactive protein, mg/L** |  |  |  | 1.00 | (0.99, 1.01) | 0.981 |
| **Sodium in urine, mmol/L** |  |  |  | 1.03 | (0.99, 1.07) | 0.090 |
| **Red blood cell count, 10^12/L** |  |  |  | 1.21 | (1.08, 1.35) | <0.001 |
| **Gamma glutamyltransferase, U/L** |  |  |  | 1.03 | (0.99, 1.07) | 0.129 |
| **Creatinine (enzymatic) in urine, mcmol/L** |  |  |  | 1.05 | (1.01, 1.09) | 0.006 |
| **Alkaline phosphatase, U/L** |  |  |  | 0.96 | (0.92, 1.00) | 0.052 |

*BrCa, Breast Cancer; HR, hazard ratio (for continuous variables, HR represents each 1 standard deviation increase); CI, confidence interval; p, p-value. Note*: Baseline model contains all established BrCa risk factors whereas New model contains established BrCa risk factors and novel features from machine learning. Alcohol intake was scaled from weekly intake to daily intake for interpretation and direct comparison with literature; Both PRS, basal metabolic rate, sodium in urine, gamma glutamyltransferase, creatinine in urine, alkaline phosphatase, and summed MET minutes per week were standardised using the mean and standard deviation within each imputed dataset, hence the corresponding HR represents per 1 standard deviation increase. For other continuous variables, HR represents per 1 unit increase. Genetic array and first 10 PCs were adjusted in the model but omitted from the table.*

The focus of our paper was to identify factors that were predictive of breast cancer by developing prediction models, which is a different task from identifying potential causal risk factors. The reason for not constructing analytical models in the previously-unseen held-out test data was that the latter were used to evaluate model performance and guard against overfitting, in keeping with conventional best-practice for model construction. The conventional usage of test data is that one would take the fixed model (with the coefficients obtained from the training data) and apply it to previously-unseen held-out data to determine how well it generalises to these new data. Therefore, fitting the model on test data would violate the purpose of the training-test split – informally, one should “fix the model” using the training set, and then use that fixed model with previously-unseen held-out data to evaluate performance objectively. (Conversely, if we retrain the model using the held-out test data, then the latter is reporting performance in-sample; that is, its performance is being reported using the same data as were used to train it, and we are not obtaining a sufficiently objective estimate of how well the model will generalise to previously-unseen data.)

We note that our approach of using training data for both feature selection and Cox model construction is common practice in the classical statistical domain. For example, one may use backward selection to choose features for inclusion in the subsequent analytical (e.g. Cox) models. That is, both feature selection and model fitting are done within the same training data. The only difference in our approach is that we used ML instead of backward selection for feature selection, which we believe makes an interesting comparison to conventional approaches – with the intention of demonstrating that ML methods can identify potentially-interesting candidate associated factors.

However, aside from the above and for completeness, we presented the results of using a Cox model with the 20% test data, and described the results in Supplementary Table 7 below.

Supplementary Table 7. Cox regression of time until breast cancer in post-menopausal women (758 events) run in test data. Baseline model represents multivariable Cox model containing two PRS and established risk factors. New model represents multivariable Cox model containing all covariates in baseline model and novel features discovered by XGBoost.

|  | Baseline model | | | New model | | |
| --- | --- | --- | --- | --- | --- | --- |
| Coefficient | HR | 95% CI | p | HR | 95% CI | p |
| $PRS_{120k}$ | 1.49 | (1.35, 1.63) | <0.001 | 1.48 | (1.35, 1.63) | <0.001 |
| $PRS_{313}$ | 1.19 | (1.08, 1.30) | <0.001 | 1.19 | (1.08, 1.30) | <0.001 |
| **Age at recruitment, years** | 1.03 | (1.01, 1.04) | <0.001 | 1.03 | (1.02, 1.05) | <0.001 |
| **BMI, kg/m^2** | 1.03 | (1.01, 1.04) | <0.001 | 1.00 | (0.98, 1.03) | 0.717 |
| **Age at menopause, years** | 1.01 | (1.00, 1.03) | 0.057 | 1.01 | (1.00, 1.03) | 0.059 |
| **Daily alcohol intake, units** | 1.03 | (0.98, 1.09) | 0.274 | 1.03 | (0.98, 1.09) | 0.286 |
| **Age at first birth, years (Categorical)** |  |  |  |  |  |  |
| No Births | 1 |  |  | 1 |  |  |
| <20 | 1.38 | (0.92, 2.07) | 0.124 | 1.39 | (0.93, 2.10) | 0.112 |
| 20-30 | 1.25 | (0.92, 1.68) | 0.150 | 1.25 | (0.93, 1.69) | 0.141 |
| 30-40 | 1.59 | (1.18, 2.16) | 0.003 | 1.60 | (1.18, 2.16) | 0.002 |
| >=40 | 2.02 | (0.93, 4.36) | 0.075 | 2.07 | (0.96, 4.47) | 0.066 |
| **Family history of BrCa** |  |  |  |  |  |  |
| No family history of BrCa | 1 |  |  | 1 |  |  |
| Family history of BrCa | 1.35 | (1.11, 1.64) | 0.003 | 1.34 | (1.10, 1.63) | 0.004 |
| **Summed MET minutes per week** | 0.97 | (0.88, 1.06) | 0.457 | 0.97 | (0.88, 1.06) | 0.467 |
| **HRT user** | 2.55 | (1.82, 3.58) | <0.001 | 2.47 | (1.74, 3.50) | <0.001 |
| **Age at menarche, years** | 0.99 | (0.94, 1.03) | 0.547 | 0.98 | (0.94, 1.03) | 0.512 |
| **IGF-1, nmol/L** | 1.01 | (0.99, 1.02) | 0.225 | 1.01 | (0.99, 1.02) | 0.239 |
| **Testosterone, nmol/L** | 1.15 | (1.04, 1.27) | 0.009 | 1.14 | (1.03, 1.26) | 0.012 |
| **Number of live births** | 0.84 | (0.76, 0.93) | 0.001 | 0.84 | (0.76, 0.93) | 0.001 |
| **Plasma urea, mmol/L** |  |  |  | 0.99 | (0.94, 1.06) | 0.848 |
| **Basal metabolic rate, KJ** |  |  |  | 1.11 | (0.99, 1.25) | 0.085 |
| **Aspartate aminotransferase, U/L** |  |  |  | 1.00 | (0.99, 1.00) | 0.336 |
| **Plasma phosphate, mmol/L** |  |  |  | 0.76 | (0.43, 1.33) | 0.339 |
| **C-reactive protein, mg/L** |  |  |  | 1.00 | (0.99, 1.02) | 0.817 |
| **Sodium in urine, mmol/L** |  |  |  | 1.01 | (0.93, 1.09) | 0.875 |
| **Red blood cell count, 10^12/L** |  |  |  | 1.07 | (0.85, 1.35) | 0.549 |
| **Gamma glutamyltransferase, U/L** |  |  |  | 1.05 | (0.97, 1.13) | 0.198 |
| **Creatinine (enzymatic) in urine, mcmol/L** |  |  |  | 1.00 | (0.92, 1.09) | 0.953 |
| **Alkaline phosphatase, U/L** |  |  |  | 1.02 | (0.94, 1.11) | 0.611 |

*BrCa, Breast Cancer; HR, hazard ratio (for continuous variables, HR represents each 1 standard deviation increase); CI, confidence interval; p, p-value. Note*: Baseline model contains all established BrCa risk factors whereas New model contains established BrCa risk factors and novel features from machine learningAlcohol intake was scaled from weekly intake to daily intake for interpretation and direct comparison with literature; Both PRS, basal metabolic rate, sodium in urine, gamma glutamyltransferase, creatinine in urine, alkaline phosphatase, and summed MET minutes per week were standardised using the mean and standard deviation within each imputed dataset, hence the corresponding HR represents per 1 standard deviation increase. For other continuous variables, HR represents per 1 unit increase. Genetic array and first 10 PCs were adjusted in the model but omitted from the table.*

# Plots

## Forest plot after excluding first 2 years follow-up

*
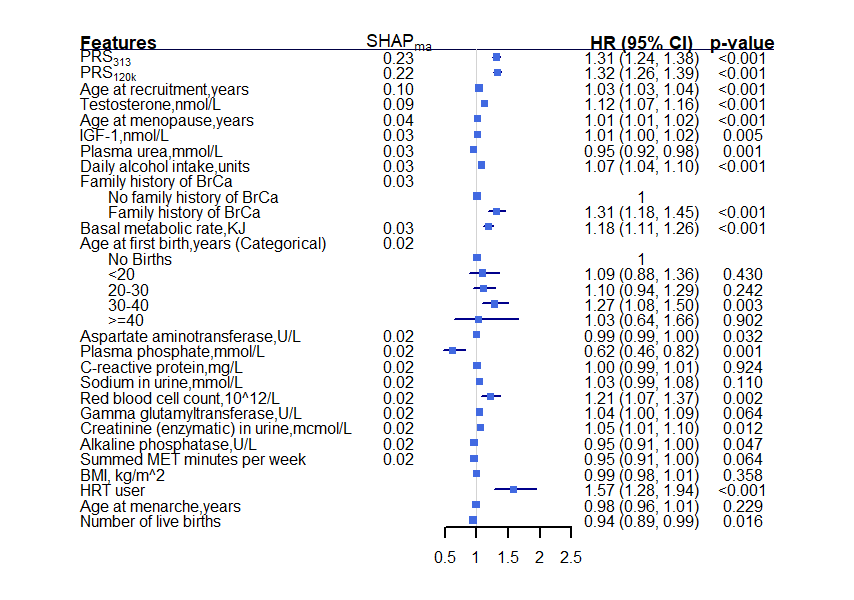
*

Supplementary Figure 4. Results obtained from the augmented multivariable Cox model after excluding first two years of follow-up using training data ranked by SHAP_ma_.The bottom four features are established risk factors that are outside the top 20 features by SHAP_ma_. Alcohol intake was scaled from weekly intake to daily intake for easy interpretation and direct comparison with existing literature. Both PRS, basal metabolic rate, sodium in urine, creatinine in urine, alkaline phosphatase, gamma glutamyltransferase, and summed MET minutes per week were standardised using the mean and standard deviation within each imputed dataset, hence the corresponding HR represents per 1 standard deviation increase. For other continuous variables, HR represents per 1 unit increase. Genetic array and first 10 PCs were adjusted in the model but omitted from the figure. SHAP: SHapley Additive explanation. SHAP_ma_: mean absolute SHAP value. BrCa: Breast Cancer. HR: hazard ratio. CI: confidence interval. HRT: hormone replacement therapy. MET: Metabolic Equivalent Task. U/L: units per litre.

## SHAP dependence plots

Our SHAP dependence plots indicated potential interactions between PRS and a range of phenotypic features (e.g. age and sodium in urine).

| 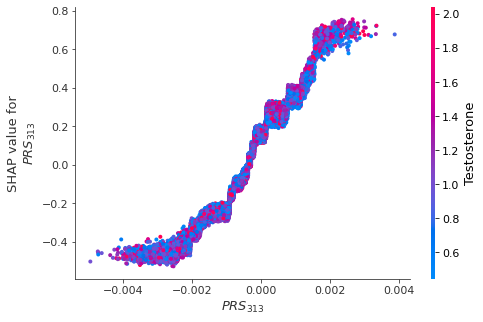  (a) | 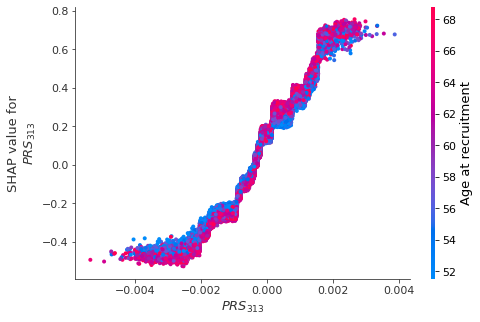  (b) |
| --- | --- |
| 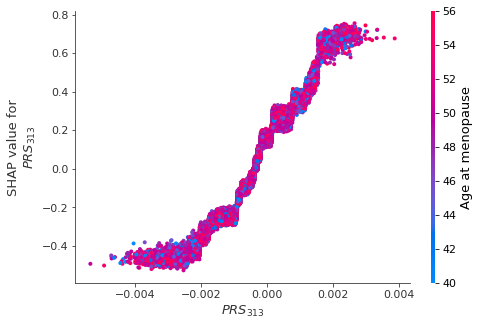  (c) | 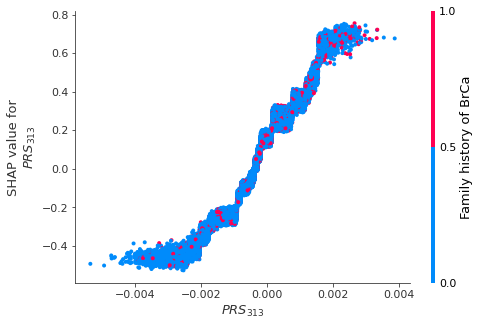  (d) |
| 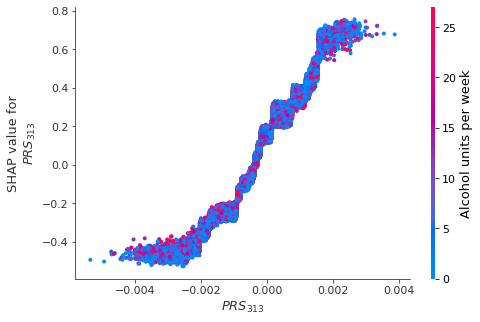  (e) | 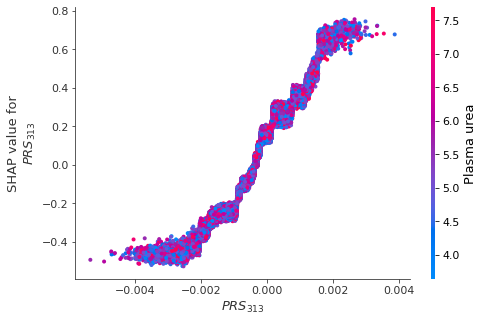  (f) |
| 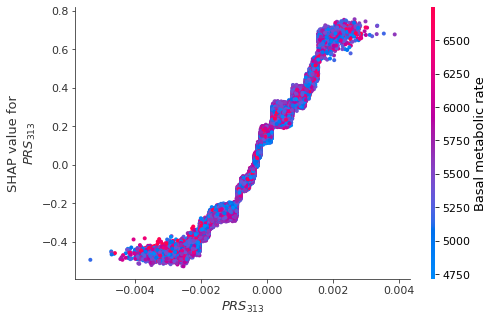  (g) | 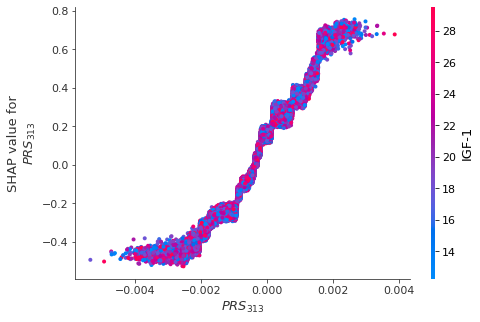  (h) |
| 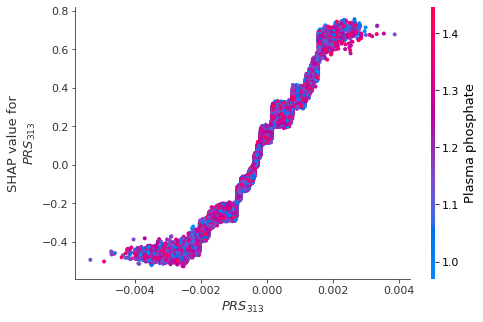  (i) | 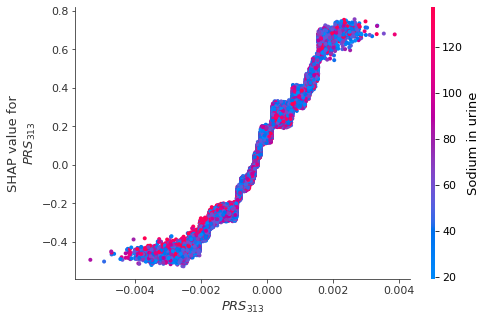  (j) |
| 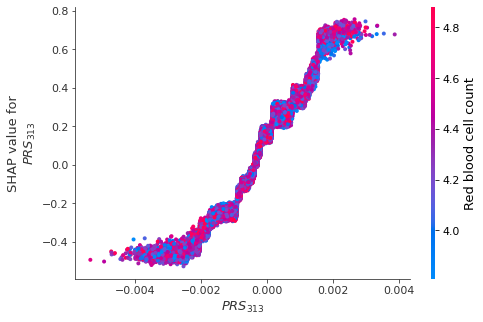  (k) | 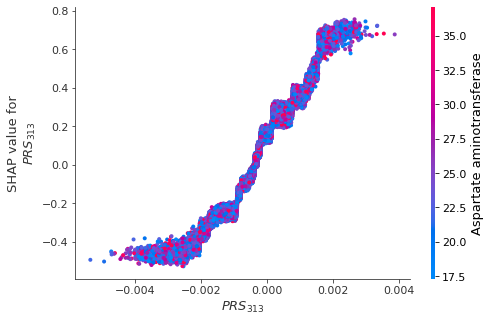  (l) |
| 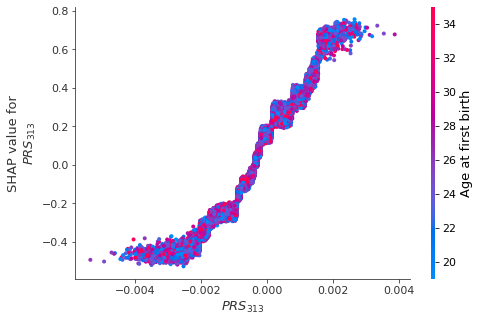  (m) | 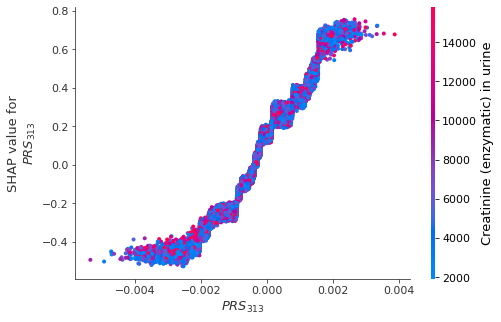  (n) |
| 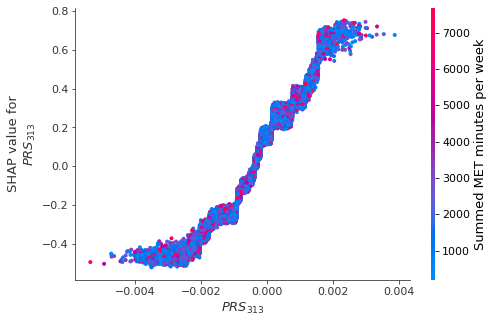 (o) | 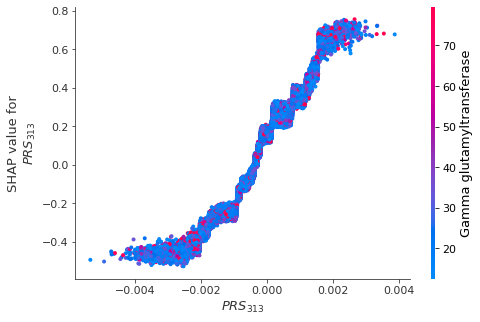  (p) |
| 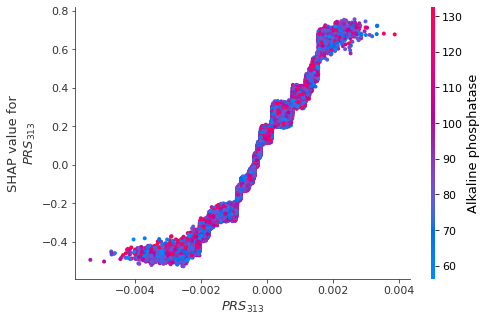  (q) | 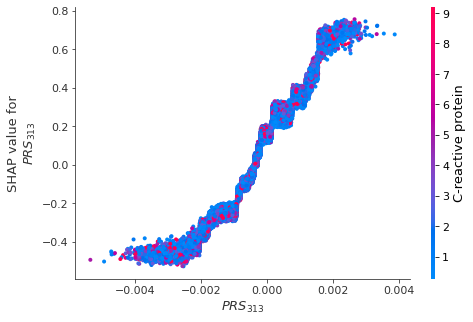  (r) |

*Supplementary Figure 5. SHAP dependence plots of PRS_313_ against the top 20 phenotypic features for breast cancer.*

| 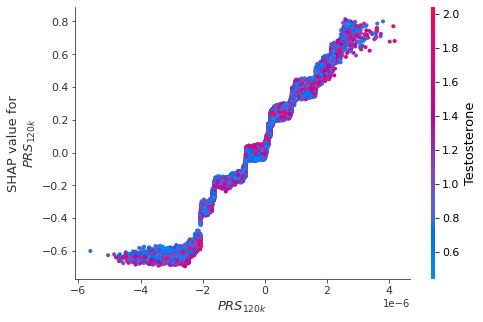  (a) | 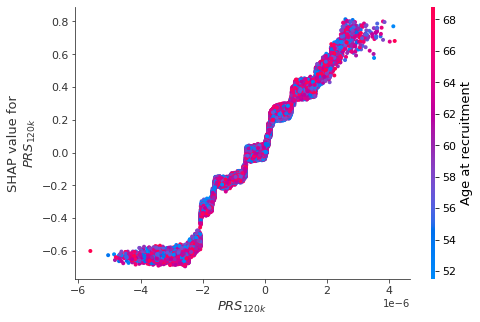  (b) |
| --- | --- |
| 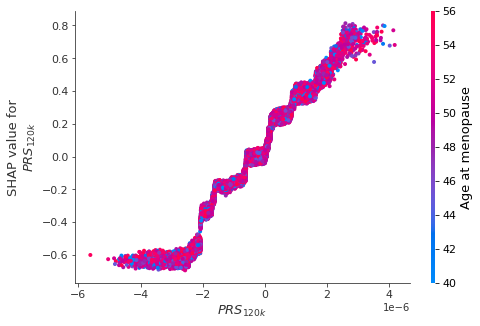 (c) | 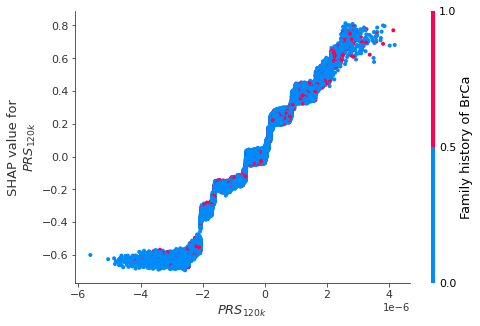  (d) |
| 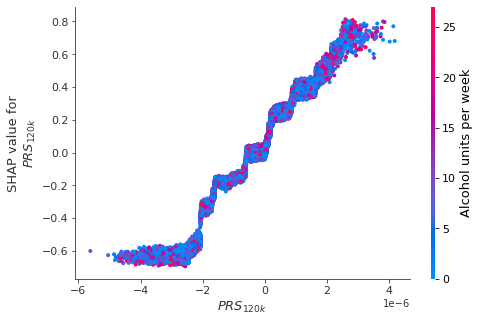 (e) | 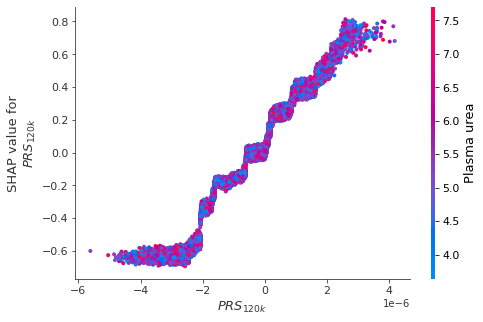  (f) |
| 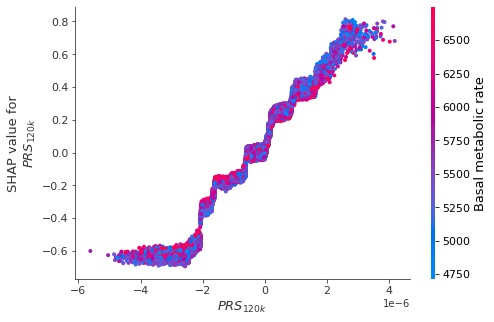  (g) | 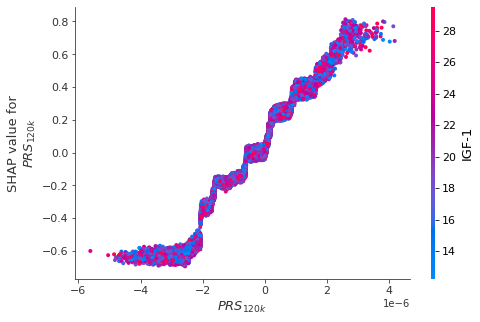  (h) |
| 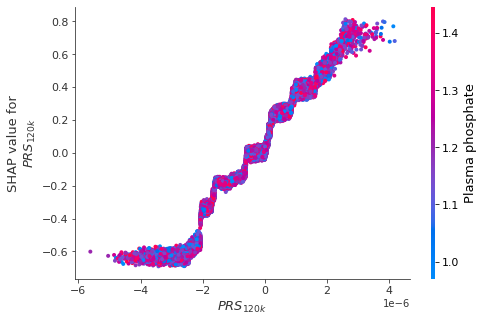 (i) | 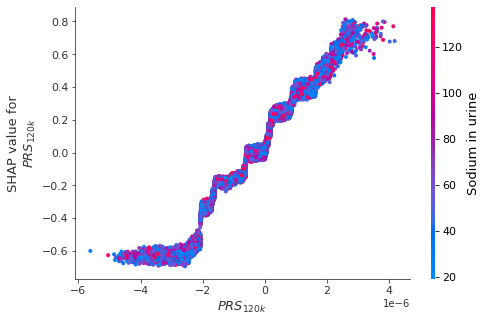  (j) |
| 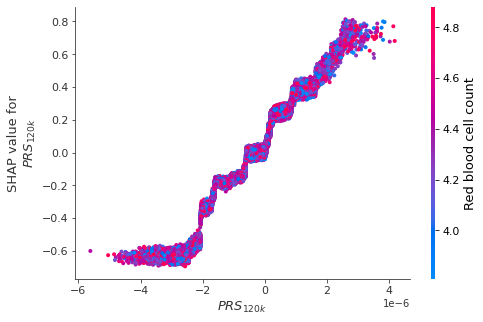 (k) | 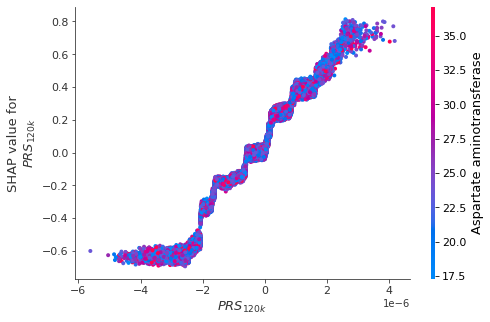  (l) |
| 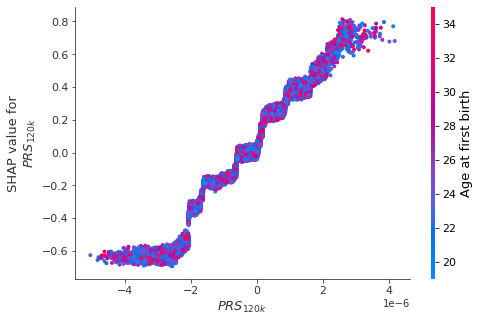  (m) | 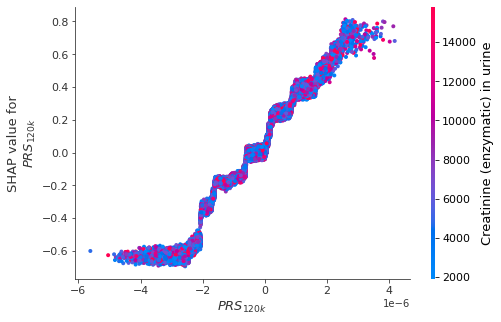  (n) |
| 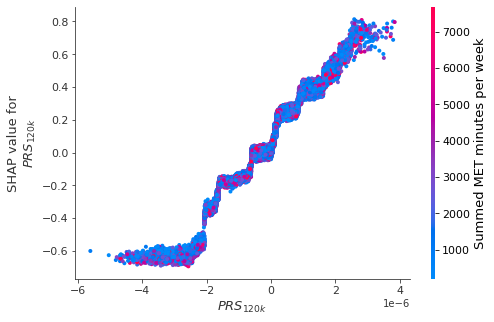 (o) | 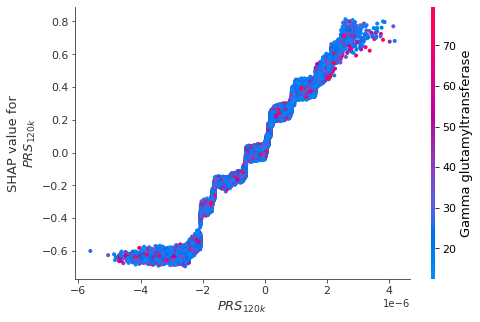 (p) |
| 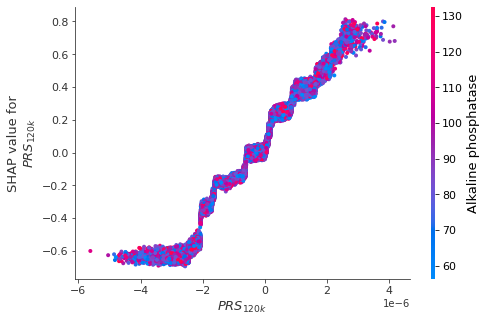  (q) | 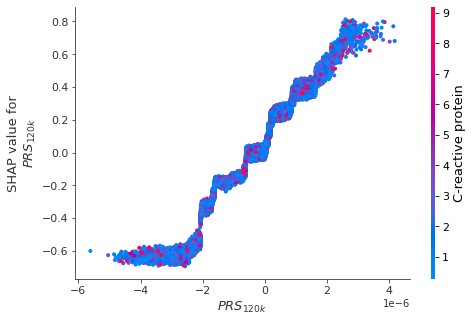  (r) |

*Supplementary Figure 6. SHAP dependence plots of PRS_120k_ against the top 20 phenotypic features for breast cancer.*

# SHAP dependence plot of PRS against age

We utilised SHAP dependence plots to identify potential interactions between PRS and potential risk factors, and subsequently constructed Cox models for in-depth investigation.

For both PRS_313_ and PRS_120k,_ SHAP dependence plots indicated potential effect modification of PRS with age and sodium in urine (Supplementary Figure 5-Supplementary Figure 6). We illustrate this in Supplementary Figure 7 which shows one of the most distinctive interactions, Age $\times$ PRS, where higher SHAP values indicate higher relative hazard of developing breast cancer due to age. Age values are colour-coded from blue to red, representing young to old.

| 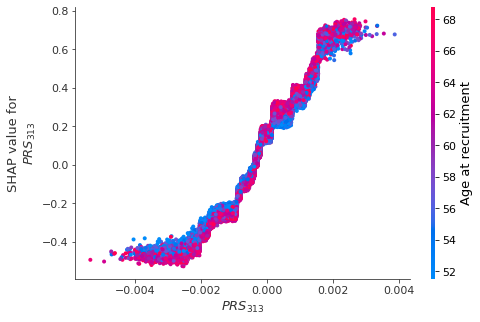 (a) | 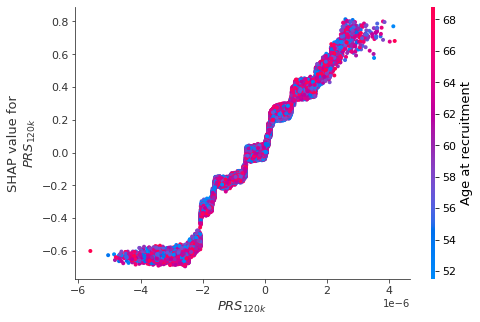 (b) |
| --- | --- |

Supplementary Figure 7. SHAP dependence plot showing PRS against age, where each dot represents each participant in the study population; (a) PRS_313_ and (b) PRS_120k_. The y-axis shows the SHAP values attributed to PRS (x-axis). Higher SHAP values indicate higher relative hazard of developing breast cancer. The corresponding age of each individual is represented by colour, with blue indicating young and red indicating old age.

Both subplots in Supplementary Figure 7 show a clear positive association of PRS on developing breast cancer where the risk of developing breast cancer increase with PRS. When taking age (represented by colour) into account, we observed a vertical dispersion of colours across PRS_313_. Such dispersion of colours suggests that age has an impact on the importance of PRS_313_ (as quantified by SHAP values). For example, young women (blue) typically had higher SHAP values for PRS_313_ than older women (red) among lower PRS_313_ values, indicating the effect of PRS_313_ was stronger on breast cancer for younger women. By contrast, the higher relative location of the red dots above the blue for higher PRS_313_ values indicates the effect of PRS_313_ was stronger for older women with higher PRS_313_. We did not observe such obvious interaction effect for PRS_120k_ with age_._

The effect modification of PRS by age indicated by the SHAP dependence plots is consistent with the longstanding literature ^10–15^ where the association between PRS and breast cancer risk was shown to decrease with age. It is worth mentioning that all these literature used simpler traditional statistical models (e.g. Cox models with Age $\times$ PRS interaction) to explore such non-linear relationships. The fact that ML models detected the same non-linear patterns highlights the power and efficiency of traditional statistical models in some settings. The unexpected patterns which arose from Supplementary Figure 7 include older age appeared to be “protective” for women with lower PRS, and the opposite for higher PRS. In the next section, we provide our investigation of these unusual patterns.

## Investigation in Cox models

As an additional exploratory analysis to explore potential interactions indicated by the SHAP dependence plots, we added “Age $\times$ PRS” interaction terms in our final multivariable Cox model. No significant effect modification of age by PRS was found ($p=0.34$ or Age $\times$ PRS_313_, $p=0.52$ for Age $\times$ PRS_120k_), but a marginal effect plot of PRS by age (Supplementary Figure 8) suggests that the effect of high PRS is slightly stronger in the older age compared to younger age. To explore further, we constructed three separate Cox models stratifying by age group: 40-53, 53-62, and 62-70 years. Our results showed higher risk of breast cancer at higher PRS values in all age groups (Supplementary Figure 9).

| 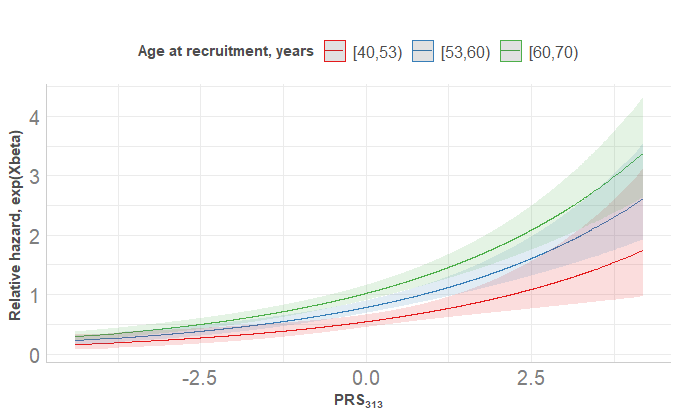  (a) | 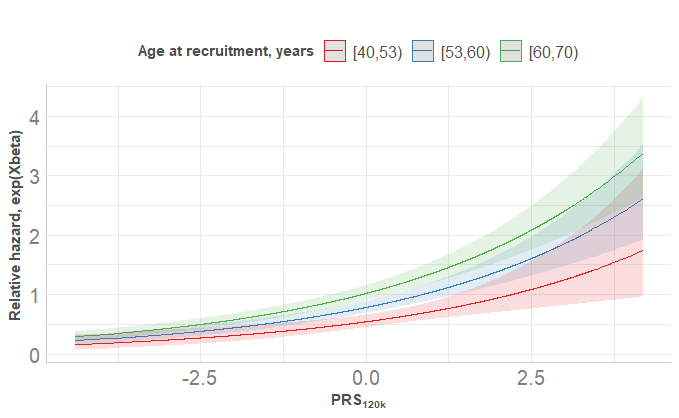  (b) |
| --- | --- |

Supplementary Figure 8. Marginal effect of age on relative hazard with pointwise 95% CI obtained from multivariable Cox model. Other continuous variables were kept as sample mean while categorical variables were kept at reference level.

| 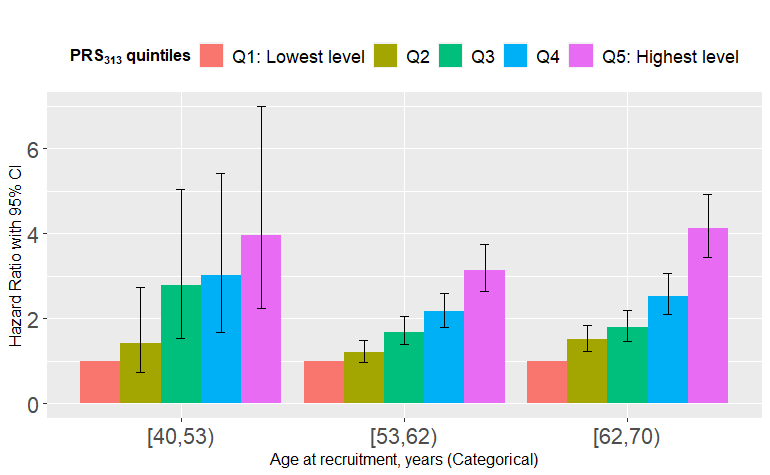  (a) | 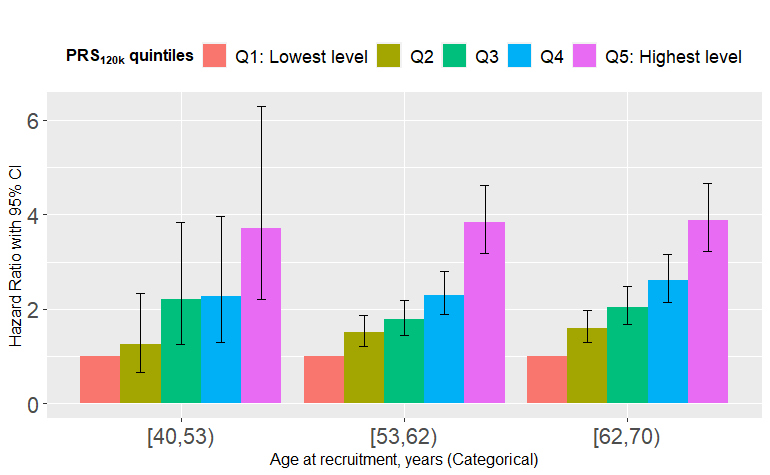  (b) |
| --- | --- |

Supplementary Figure 9. Hazard ratio with 95% confidence interval of PRS obtained from three separate Cox models stratified by age groups: [40,53), [53,62), [62,70) years, with adjustment of genetic array and first 10 PCs; (a) PRS_313_ and (b) PRS_120k_. First quintile of PRS, “Q1: Lowest level” was regarded as the reference level for comparison across other quintiles within each age group.

In summary, the interaction of Age $\times$ PRS was observed in the SHAP dependence plots, but it was not statistically significant in the subsequent Cox models.

# Exemplar code

Below demonstrates structure of our analysis code. For complete scripts, please see <https://github.com/xiaonanl1996/MLforBrCa>.

## Data derivation

We derived all relevant variables using internally developed database system (see [here](https://github.com/2cjenn/UKB_database) for database system). All scripts used for data derivation are stored under “Scripts/Data Management” (see [here](https://github.com/xiaonanl1996/MLforBrCa/blob/main/Scripts/Data_Management/README.md) for folder structure). The main script is “Scripts/Data Management/Scripts_Processing/InitialExtraction.Rmd”. Below we copied over the key part for illustration.

We used derive_variables function to derive the variables needed in the analyses (e.g. breast cancer outcomes and all input features). Variable database specifies the file path of UKB database, field_definitions specifies the list of variables one wish to derive, and exclusions specifies the function of exclusion criteria.


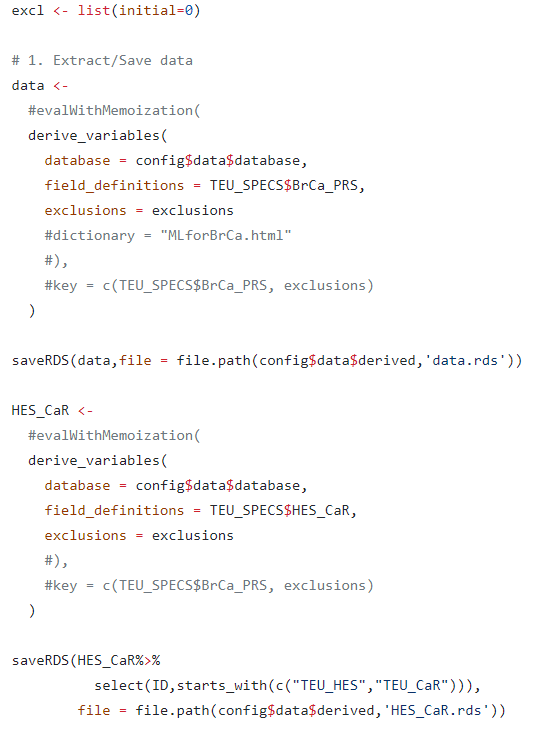


Then the chunk below produces the analysis population flowchart (i.e. Figure 1).


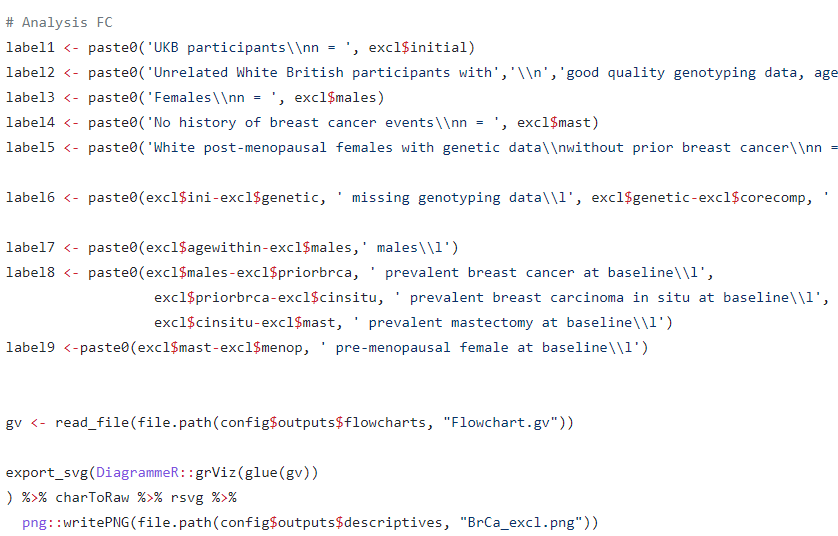


Then we split the data into training and test data, followed by pre-processing on each dataset separately.


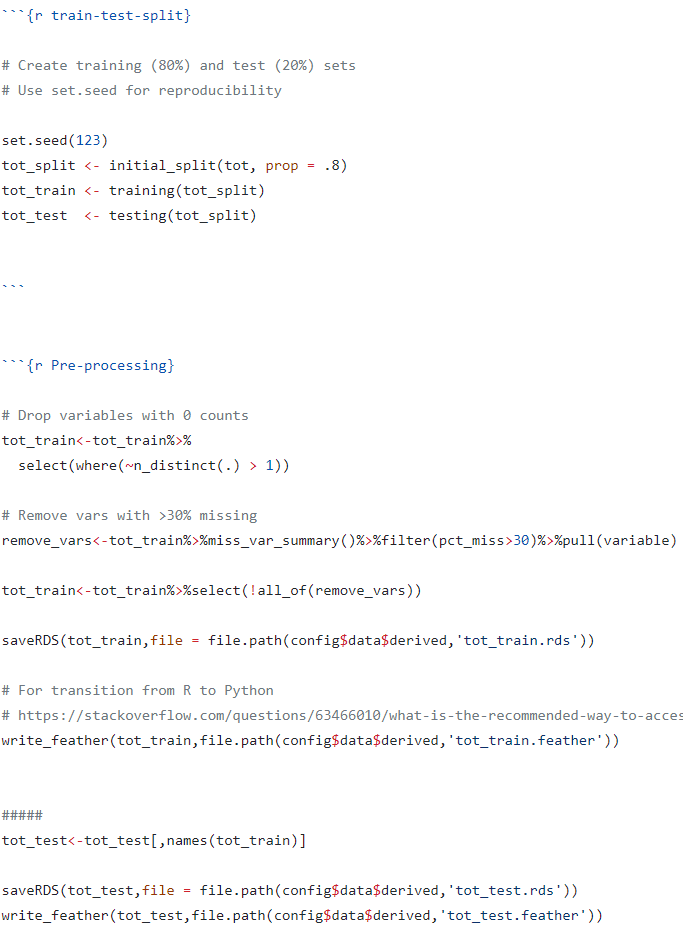


## Baseline characteristics

Knit “Scripts/Stats_Analysis/RMarkdown/MLforBrCa_BaselineTable.Rmd”. Used arsenal R package.


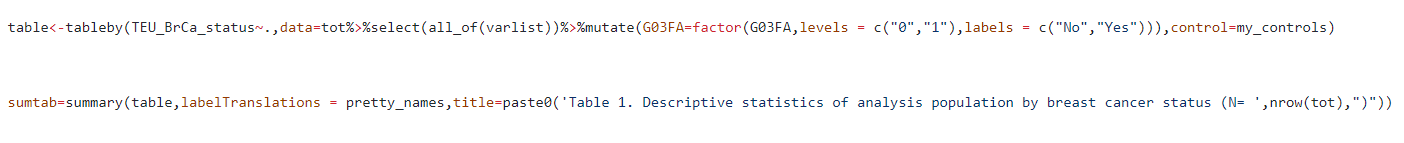


## XGBoost

The analysis script for implementing XGBoost with Cox loss is “Scripts/Stats_Analysis/JupyterNotebook/XGBoost-Cox.ipynb”.

After grid search, we fit the optimal XGBoost model using [XGBoost Python package](https://xgboost.readthedocs.io/en/stable/python/index.html):


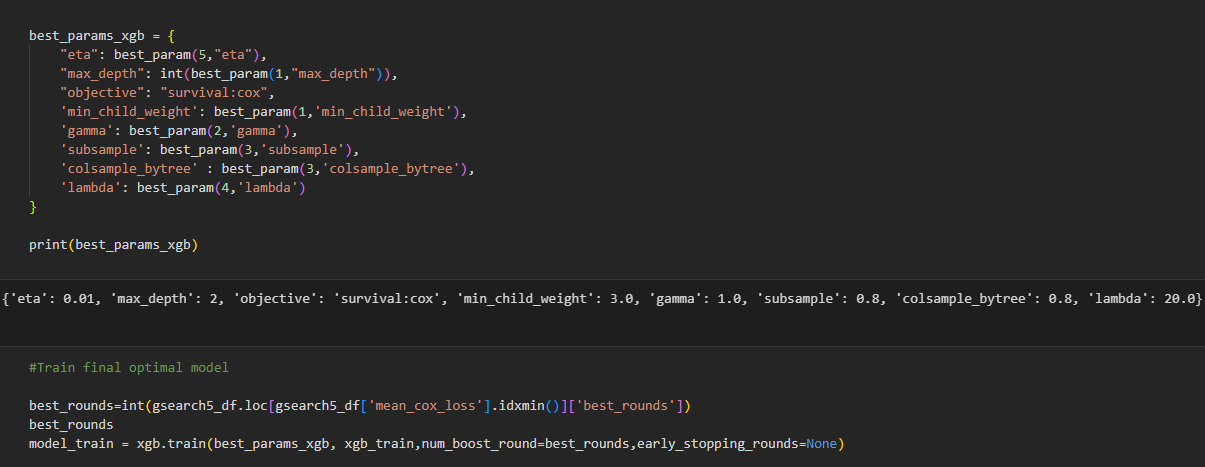


Then SHAP summary plot and beeswarm plot (see Figure 4).


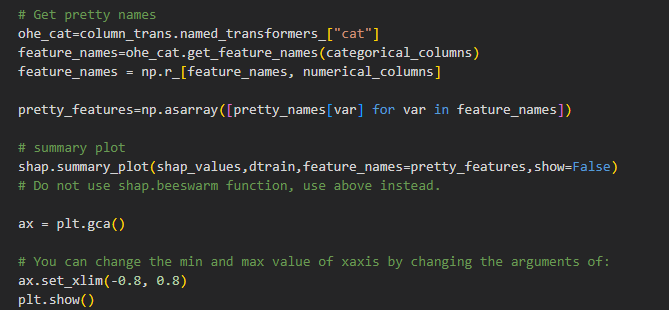


## Correlation filtering and multiple imputation

See “Scripts/Stats_Analysis/RMarkdown/Multiple_Imp.Rmd”. We used mice R package for multiple imputation.


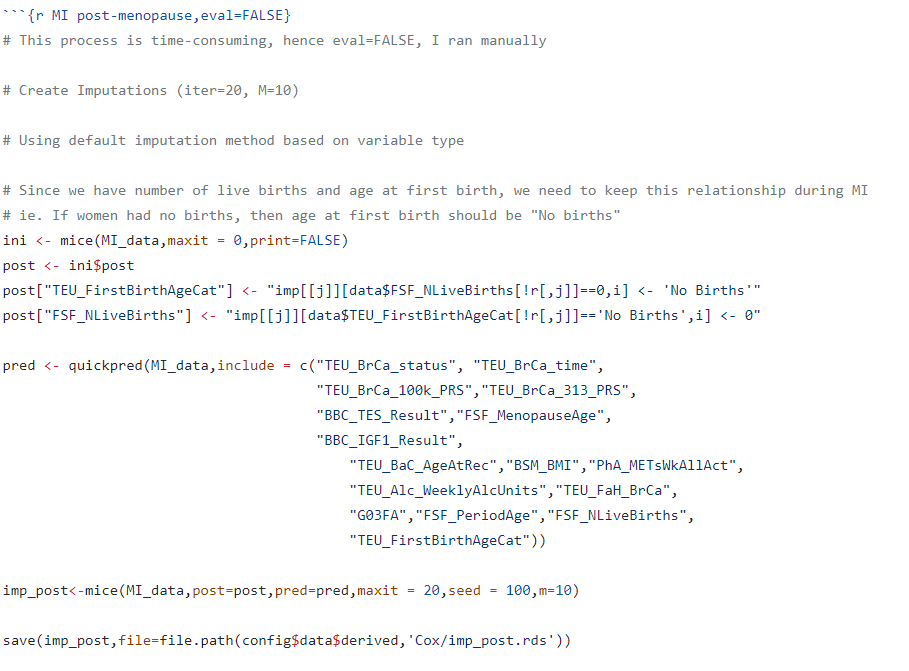


## Cox model (forest plot)

See “Scripts/Stats_Analysis/RMarkdown/MLforBrCa_CoxTable.Rmd”. We wrote a function printMIresults() (stored in “Scripts/Stats_Analysis/JCfunctions.R”) to show pretty output of Cox models. We then used forestplot R package to produce the forest plot.


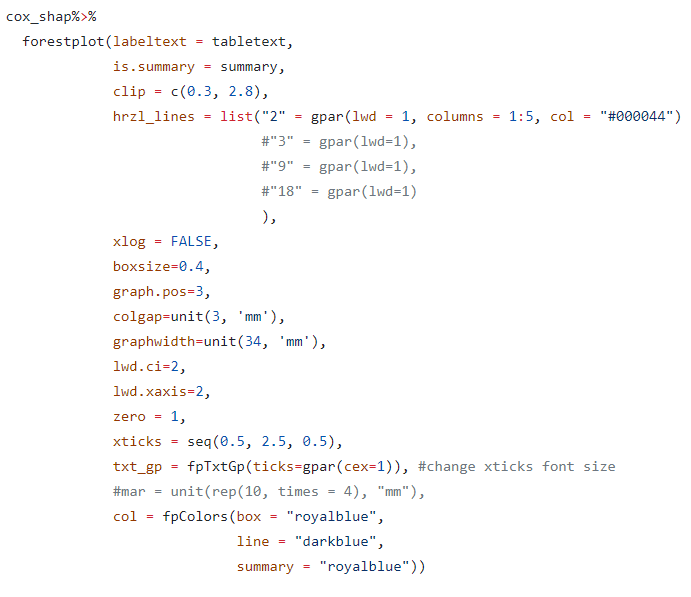


## Model performance

See “Scripts/Stats_Analysis/RMarkdown/MLforBrCa_ModelPerformance.Rmd”.

## Sensitivity analyses

See “Scripts/Stats_Analysis/JupyterNotebook/HistGBM.ipynb” and “Scripts/Stats_Analysis/RMarkdown/MLforBrCa_SA.Rmd”.

# References

1. Collister, J. A., Liu, X. & Clifton, L. Calculating Polygenic Risk Scores (PRS) in UK Biobank: A Practical Guide for Epidemiologists. *Front. Genet.* **0**, 105 (2022).

2. Larochelle, H., Erhan, D., Courville, A., Bergstra, J. & Bengio, Y. An empirical evaluation of deep architectures on problems with many factors of variation. *ACM Int. Conf. Proceeding Ser.* **227**, 473–480 (2007).

3. Hinton, G. A Practical Guide to Training Restricted Boltzmann Machines. (2010).

4. Bergstra, J., Ca, J. B. & Ca, Y. B. Random Search for Hyper-Parameter Optimization Yoshua Bengio. *J. Mach. Learn. Res.* **13**, 281–305 (2012).

5. Snoek, J., Larochelle, H. & Adams, R. P. Practical Bayesian Optimization of Machine Learning Algorithms. *Adv. Neural Inf. Process. Syst.* **25**, (2012).

6. Shapley, L. S. 17. A Value for n-Person Games. *Contrib. to Theory Games (AM-28), Vol. II* 307–318 (1953) doi:10.1515/9781400881970-018/HTML.

7. Lundberg, S. M. & Lee, S. I. A Unified Approach to Interpreting Model Predictions. *Adv. Neural Inf. Process. Syst.* **2017**-**Decem**, 4766–4775 (2017).

8. Lundberg, S. M. *et al.* From local explanations to global understanding with explainable AI for trees. *Nat. Mach. Intell. 2020 21* **2**, 56–67 (2020).

9. Ke, G. *et al.* LightGBM: A Highly Efficient Gradient Boosting Decision Tree. *Adv. Neural Inf. Process. Syst.* **30**, (2017).

10. Gao, C. *et al.* Risk of Breast Cancer Among Carriers of Pathogenic Variants in Breast Cancer Predisposition Genes Varies by Polygenic Risk Score. *J. Clin. Oncol.* **39**, 2564–2573 (2021).

11. Gallagher, S. *et al.* Association of a Polygenic Risk Score With Breast Cancer Among Women Carriers of High- and Moderate-Risk Breast Cancer Genes. *JAMA Netw. open* **3**, (2020).

12. Mavaddat, N. *et al.* Prediction of breast cancer risk based on profiling with common genetic variants. *J. Natl. Cancer Inst.* **107**, (2015).

13. Aschard, H., Zaitlen, N., Lindström, S. & Kraft, P. Variation in predictive ability of common genetic variants by established strata: the example of breast cancer and age. *Epidemiology* **26**, 51–58 (2015).

14. Lee, A. *et al.* BOADICEA: a comprehensive breast cancer risk prediction model incorporating genetic and nongenetic risk factors. *Genet. Med.* **21**, (2019).

15. Mavaddat, N. *et al.* Polygenic Risk Scores for Prediction of Breast Cancer and Breast Cancer Subtypes. *Am. J. Hum. Genet.* **104**, 21–34 (2019).
